# Host individual and gut location are more important in gut microbiota community composition than temporal variation in the marine herbivorous fish *Kyphosus sydneyanus*

**DOI:** 10.1186/s12866-023-03025-2

**Published:** 2023-09-29

**Authors:** Alessandro Pisaniello, Kim M. Handley, W. Lindsey White, Esther R. Angert, Jian Sheng Boey, Kendall D. Clements

**Affiliations:** 1 School of Biological Sciences, University of Auckland, Private Bag 92019, Auckland, New Zealand; 2 School of Science, Auckland University of Technology, Private Bag 92006, Auckland, New Zealand; 3 Department of Microbiology, Cornell University, 123 Wing Drive, Ithaca, NY 14853 USA

**Keywords:** Gut microbiota, Herbivory, Host-individual variation, Seasons, ddPCR, Community assembly

## Abstract

**Background:**

Gut microbiota play a key role in the nutrition of many marine herbivorous fishes through hindgut fermentation of seaweed. Gut microbiota composition in the herbivorous fish *Kyphosus sydneyanus* (family Kyphosidae) varies between individuals and gut sections, raising two questions: (i) is community composition stable over time, especially given seasonal shifts in storage metabolites of dietary brown algae, and (ii) what processes influence community assembly in the hindgut?

**Results:**

We examined variation in community composition in gut lumen and mucosa samples from three hindgut sections of *K. sydneyanus* collected at various time points in 2020 and 2021 from reefs near Great Barrier Island, New Zealand. 16S rRNA gene analysis was used to characterize microbial community composition, diversity and estimated density. Differences in community composition between gut sections remained relatively stable over time, with little evidence of temporal variation. Clostridia dominated the proximal hindgut sections and Bacteroidia the most distal section. Differences were detected in microbial composition between lumen and mucosa, especially at genus level.

**Conclusions:**

High variation in community composition and estimated bacterial density among individual fish combined with low variation in community composition temporally suggests that initial community assembly involved environmental selection and random sampling/neutral effects. Community stability following colonisation could also be influenced by historical contingency, where early colonizing members of the community may have a selective advantage. The impact of temporal changes in the algae may be limited by the dynamics of substrate depletion along the gut following feeding, i.e. the depletion of storage metabolites in the proximal hindgut. Estimated bacterial density, showed that Bacteroidota has the highest density (copies/mL) in distal-most lumen section V, where SCFA concentrations are highest. Bacteroidota genera *Alistipes* and *Rikenella* may play important roles in the breakdown of seaweed into useful compounds for the fish host.

**Supplementary Information:**

The online version contains supplementary material available at 10.1186/s12866-023-03025-2.

## Background

The importance of host-associated gut microorganisms to the evolution of vertebrates is well documented [1, 2]. Gut microbial communities contribute to numerous key functions in vertebrates, including facilitating immune response, vitamin biosynthesis, nitrogen recycling, detoxification and digestion [3, 4]. Community assembly in these communities is driven by various processes: environmental selection, historical contingency, ecological drift and dispersal limitation all influence the network of interactions within the host gut microbiome [5, 6]. In some vertebrates, seasonal changes in diet drive variation in the gut microbial populations [7–9]. In some herbivorous animals these seasonal changes are associated with changes in the carbohydrate composition of dietary plants [7–9].

There has been a considerable amount of work describing gut microbiomes in mammals [1, 10], including humans [11], but gut microbiomes in other vertebrates are less well understood, especially fish [10]. Recent studies have investigated gut microbiota in a large number of freshwater [12] and marine fish [13], Prevalent bacterial taxa in marine fish gut communities include the phyla Bacillota, Bacteroidota and Pseudomonadota [14–19] (formerly Firmicutes, Bacteroidetes and Proteobacteria [20]). The gut microbial communities of fish are influenced by a variety of factors including host phylogeny [13] gut location [15, 18, 21], water temperature [22], ontogeny [18, 23], diet [24–26], geographic location [16, 27] and season [25, 26, 28]. Identifying the processes that influence community assembly in the gut of marine fish is challenging because of the apparent lack of capacity for vertical transmission in most species [29] and the ability of marine fish to disperse over great distances as planktonic larvae [30]. The diversity of the gut microbiome can vary with trophic level [31, 32], however direct associations between trophic levels and diversity are still debated [13]. All fishes can readily digest and assimilate protein and lipid, but many herbivorous fishes rely on their gut microbiome to salvage energy from refractory carbohydrates of dietary seaweed [3, 32].

Gut microbiota play an important role in the nutrition of many marine herbivorous fish [3, 33, 34]. Bacteria breakdown refractory polysaccharides of seaweed and ferment sugars and the sugar alcohol mannitol to short-chain fatty acids (SCFA), thus salvaging energy for host fish [35, 36]. The sugar alcohol mannitol is the primary photosynthate of phaeophytes, and therefore is a major component of the diet of marine herbivorous fish that target these algae [37]. The total mannitol content of phaeophyte fronds can be as high as 50% dry weight [38], making them an important source of energy for fish that eat these algae [37]. The percentage of dry weight of the sugar alcohol mannitol and the storage glucan laminarin in phaeophytes vary across seasons, and typically levels are highest in summer and autumn and lowest in winter and spring [39, 40]. This raises the question whether seasonal variation in mannitol and laminarin content in dietary phaeophytes could influence the hindgut microbial community in fish that eat phaeophytes, e.g. odacine labrids [41], nasiine unicornfishes [24] and several kyphosid chubs [42, 43].

Herbivorous *Kyphosus* species (family Kyphosidae) have among the highest levels of SCFA recorded in fishes, indicating the importance of hindgut fermentation to nutrition in these fishes [15, 34, 36, 42, 44]. Silver drummer (*Kyphosus sydneyanus*) occur on rocky reefs in northern New Zealand and temperate Australia [45, 46]. Adults of *K. sydneyanus* in New Zealand have a diet dominated throughout the year by phaeophytes (i.e., *Ecklonia radiata* and *Carpophyllum maschalocarpum*) [46], and a long gastrointestinal tract with a large hindgut volume compared to other carnivorous and omnivorous species [36, 47]. Despite the process of hindgut fermentation being central to digestion and assimilation of nutrients in this species, distal gut communities exhibit high levels of variation among host individuals [15, 17]. Although the high variability among the gut microbiome of host individuals, and the temporal variability of seaweed storage metabolites, little is currently known about temporal stability of the hindgut microbiome, or how the communities are assembled.

The present study examined variation across multiple sampling time points in taxonomic gut microbial community composition (using the 16S rRNA gene) and microbial density in adult *K. sydneyanus* collected from subtidal reefs in north-eastern New Zealand. Our study addresses two main questions: (i) is community composition stable across time, especially given seasonal shifts in storage metabolites of dietary brown algae, and (ii) what processes influence community assembly in the hindgut?

## Results

### Microbiota composition across gut sections, gut locations (lumen and mucosa) and time

**Fig. 1:**
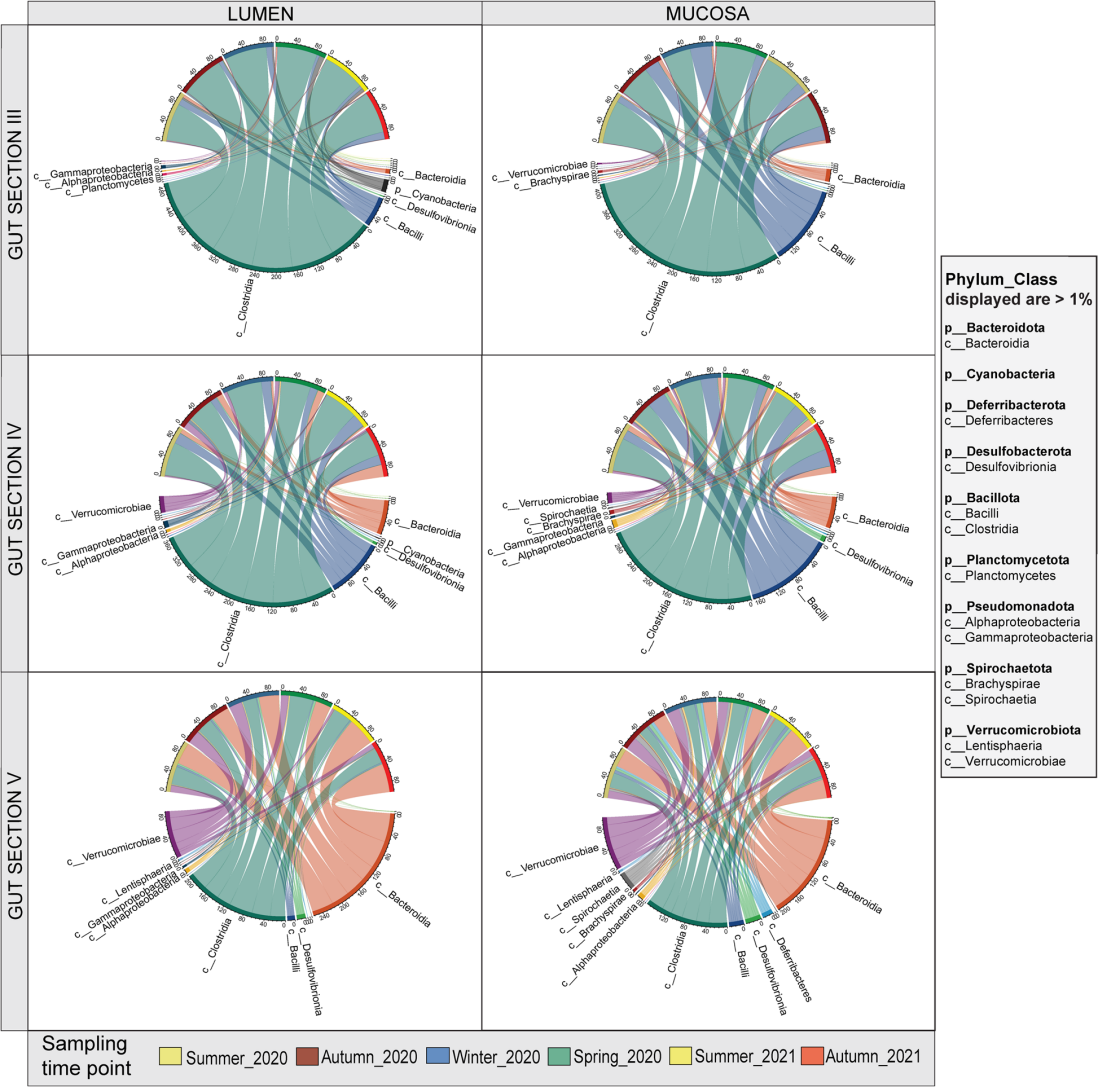
Plots showing the composition and relative abundance of hindgut microbial communities in sections III, IV and V. Relative abundance of lumen (left circle plots) and mucosa (right circle plots) composition at class level. Top ring colours indicate sampling time points (summer 2020 to autumn 2021). Sampling time points are indicated as seasons. Taxa phyla and class names are given for each class present at least > 1% relative abundance on average in one taxon

The relative abundance of bacterial community composition for hindgut sections and locations (lumen and mucosa) across time was determined by 16S rRNA gene sequence analysis. The gut microbiota was dominated by the classes Clostridia (phylum Bacillota) and Bacteroidia (phylum Bacteroidota) (Fig. 1 and Supplementary Fig. 1), followed by less abundant taxa including Bacilli (phylum Bacillota), Verrucomicrobiae (phylum Verrucomicrobiota) and Spirochaetia (phylum Spirochaetota). Bacillota and Bacteroidota both displayed temporal variation in sections IV and V, but there were no clear or consistent trends through time (e.g. consistent increases or decreases in relative abundance) (Supplementary Fig. 1). Gut microbiota did not vary temporally across the six study seasonal time points in terms of either composition or relative abundance (Fig. 1).

Clostridia were the most abundant class in gut sections III and IV of both lumen and mucosa and across all sampling time points. The mean relative read abundance (RRA) (± SE) of Clostridia in hindgut section III across time points was between 75 ± 6.5% and 86 ± 4.2% in the lumen (minimum, summer 2020; maximum, autumn 2020), and between 53 ± 3.4% and 84 ± 6.8% in the mucosa (minimum, winter 2020; maximum, summer 2020). In gut section IV, mean Clostridia RRA ± SE was between 50 ± 8.8% and 69 ± 3.1% in the lumen (minimum, autumn 2021; maximum, winter 2020), and between 39% ± 3.3% and 65% ± 5.0% in the mucosa (minimum, autumn 2020; maximum, summer 2020). While Clostridia was most abundant in gut sections III and IV, Bacteroidia dominated section V across all time points with mean RRA ± SE between 30% ± 0.1 and 56% ± 0.6 (minimum, spring 2020; maximum, autumn 2021) in the lumen and between 26% ± 0.6 and 48% 1.1 ± SE (minimum, winter 2020; maximum, summer 2021) in the mucosa (Fig. 1). Redundancy discriminant analysis (RDA) of ASVs, and Microbiome Multivariable Association with Linear Models (MaAsLin2) analysis, which was used to test significant multivariable associations, show similar results as observed in Fig. 1, i.e. Bacillota (Clostridia) had the strongest effect in section III and Bacteroidota in section V (Supplementary Figs. 2 and 3).

The community composition of gut microbiota appeared broadly similar between lumen and mucosa, however slight differences were detected. The mean RRA of Bacilli in the mucosa section III was up to 41% ± 3.3% (maximum, winter 2020), while in lumen it was only up to 14% ± 3.9% (maximum, autumn 2021). Verrucomicrobiota was present at similar proportions in both lumen and mucosa communities within gut sections IV and V and across sampling time points. However, when comparing gut sections, it was almost absent in section III. Verrucomicrobiota also increased slightly within the hindgut (i.e. in section V). We also found that Spirochaetia in section V mucosa contributed up to 10% of some communities (autumn 2020), while it was close to 0% in the lumen across all the time points (Fig. 1).

**Fig. 2:**
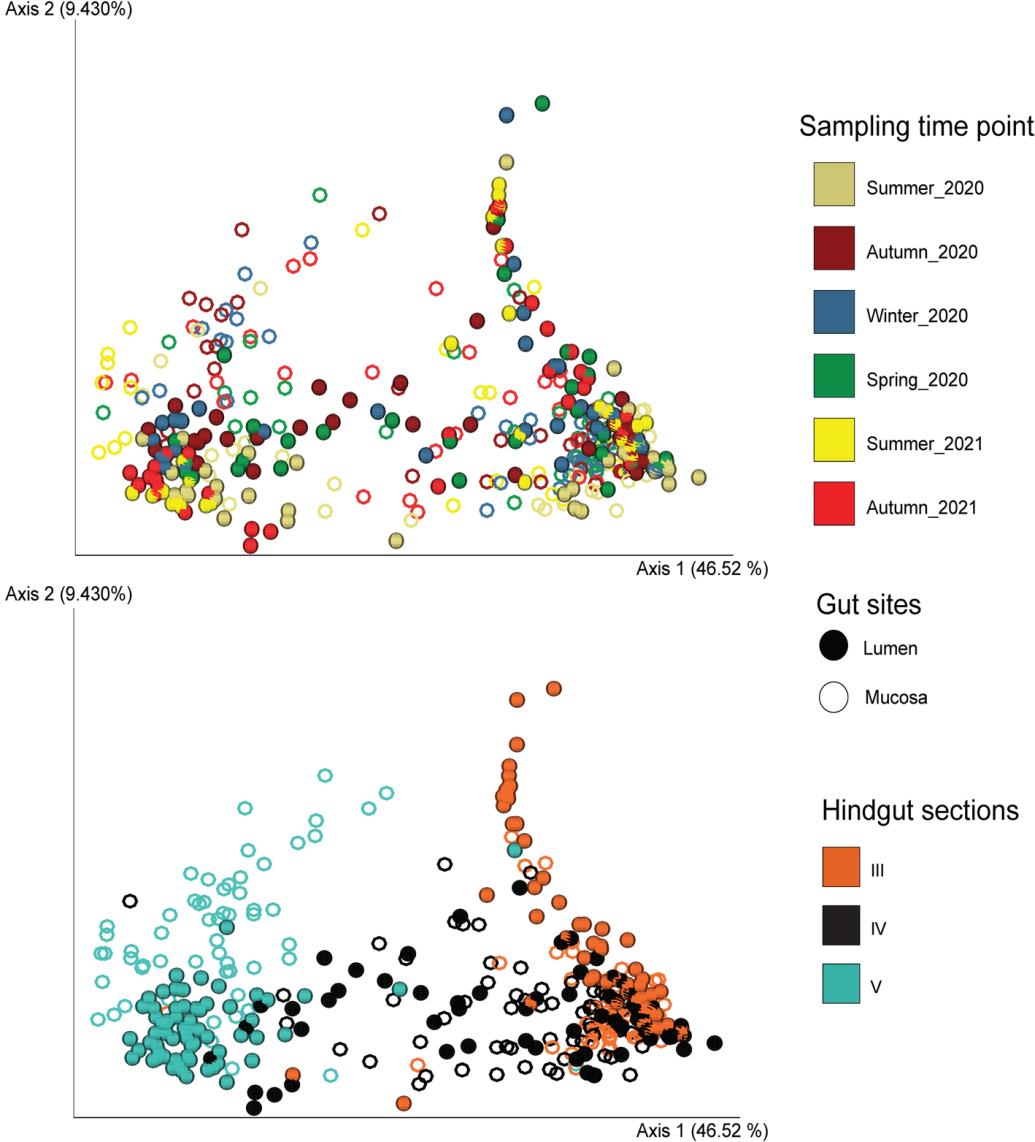
Weighted UniFrac PCoA of lumen and mucosa samples across sampling time points (top plot) and hindgut sections (bottom plot)

**Fig. 3:**
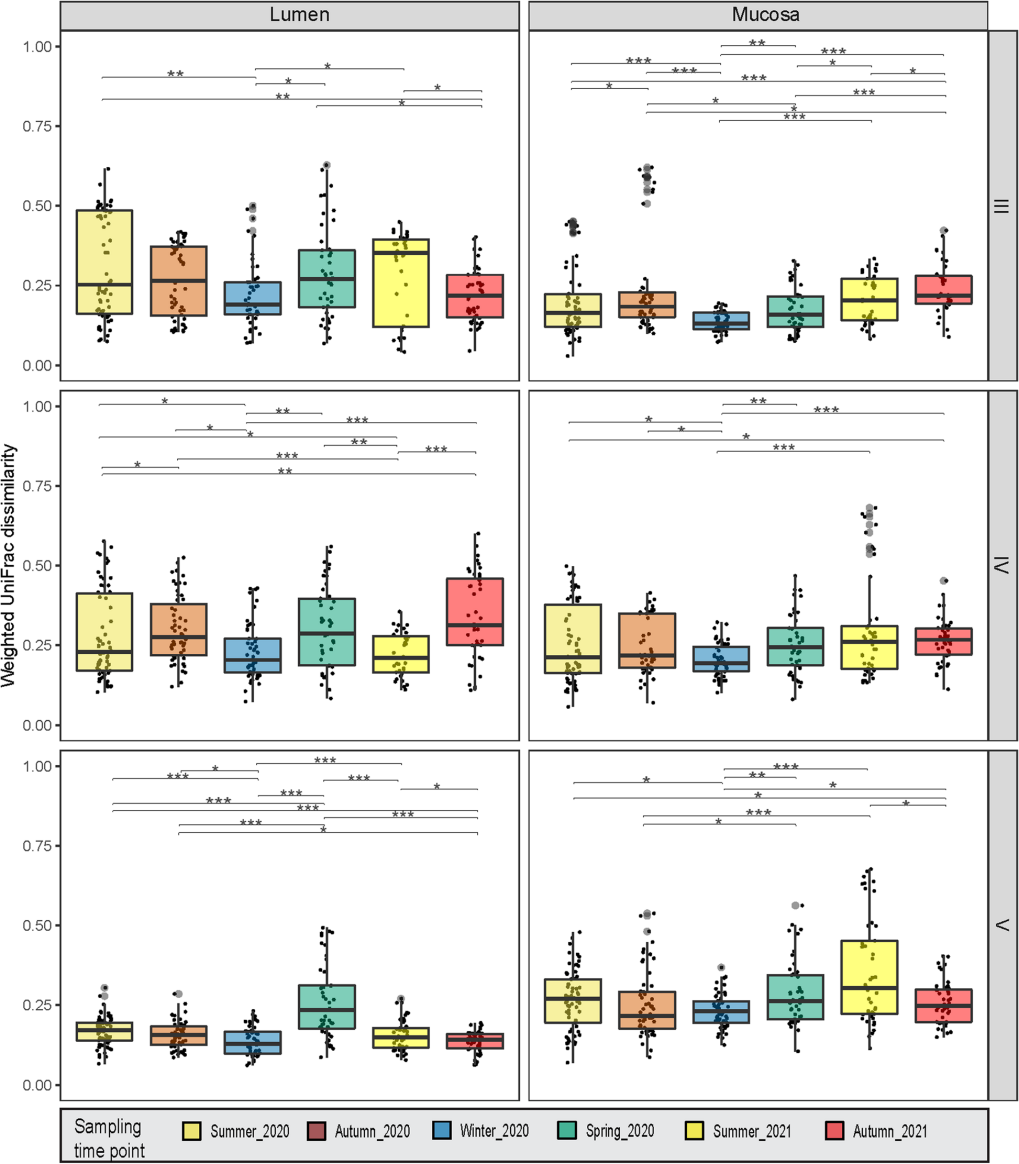
Weighted UniFrac dissimilarity within sampling time points across gut sections and between lumen and mucosa. All boxplot distributions are tested by a non-parametric Kruskal–Wallis (KW) with a post-hoc Dunn test

Weighted UniFrac PCoA analysis of microbial community beta diversity (top plot in Fig. 2, and Supplementary Fig. 4) and RDA (Supplementary Fig. 5a) further showed a lack of variation among sampling time points. However, the PERMANOVA results of PCoA (Table 1) revealed significant differences between time points, both when considering communities overall and within individual hindgut sections (PERMANOVA, *p*-value < 0.001). This indicates some variation in community composition between collection time points. Bacterial community composition also differed between lumen and mucosa within all gut sections (PERMANOVA, *p*-value < 0.001). Microbiota samples clustered by hindgut section (PERMANOVA, *p*-value < 0.001), indicating the presence of distinct microbial community composition along the hindgut of *K. sydneyanus* (Fig. 2 bottom and Table 1). Similarly, ANOVA results of RDA considering temperature (quantitative environmental variable for collection time points), gut sites and gut sections (categoric environmental variables) resulted in *p*-values < 0.001 (Supplementary Fig. 5b and Supplementary Table 1).To investigate this variation between sampling time points further we plotted Weighted UniFrac dissimilarities for each time point, gut section and location (Fig. 3). Significant Kruskal–Wallis *p*-values indicate significant differences between the 2020 and 2021 summer and autumn samples, suggesting that the differences are due to variation at the level of individual fish collected at each time point, not between the sampling time points themselves. However, unmeasured temporal factors may also account for at least some of the variation among individuals.

**Table 1:** Permutational multivariate analysis of variance (PERMANOVA) of gut sections and sampling time points in lumen and mucosa

Source of variation		Df	Sum of Sqs	R2	F	Pr(> F)
Gut sites (lumen vs mucosa)		1	0.750	0.0217	8.3396	0.001
Lumen	Hindgut sections (III, IV, V)	2	8.9448	0.52017	125.4761	0.001
	Section × sampling time points	10	1.0187	0.05924	2.8581	0.001
	Sampling time points	5	1.1374	0.06614	6.3819	0.001
Mucosa	Hindgut sections (III, IV, V)	2	8.4130	0.50574	118.0542	0.001
	Section × sampling time points	10	0.9111	0.05477	2.5569	0.001
	Sampling time points	5	1.2180	0.07322	6.8367	0.001

### Alpha diversity and genera composition of gut sections and between lumen and mucosa

**Fig. 4:**
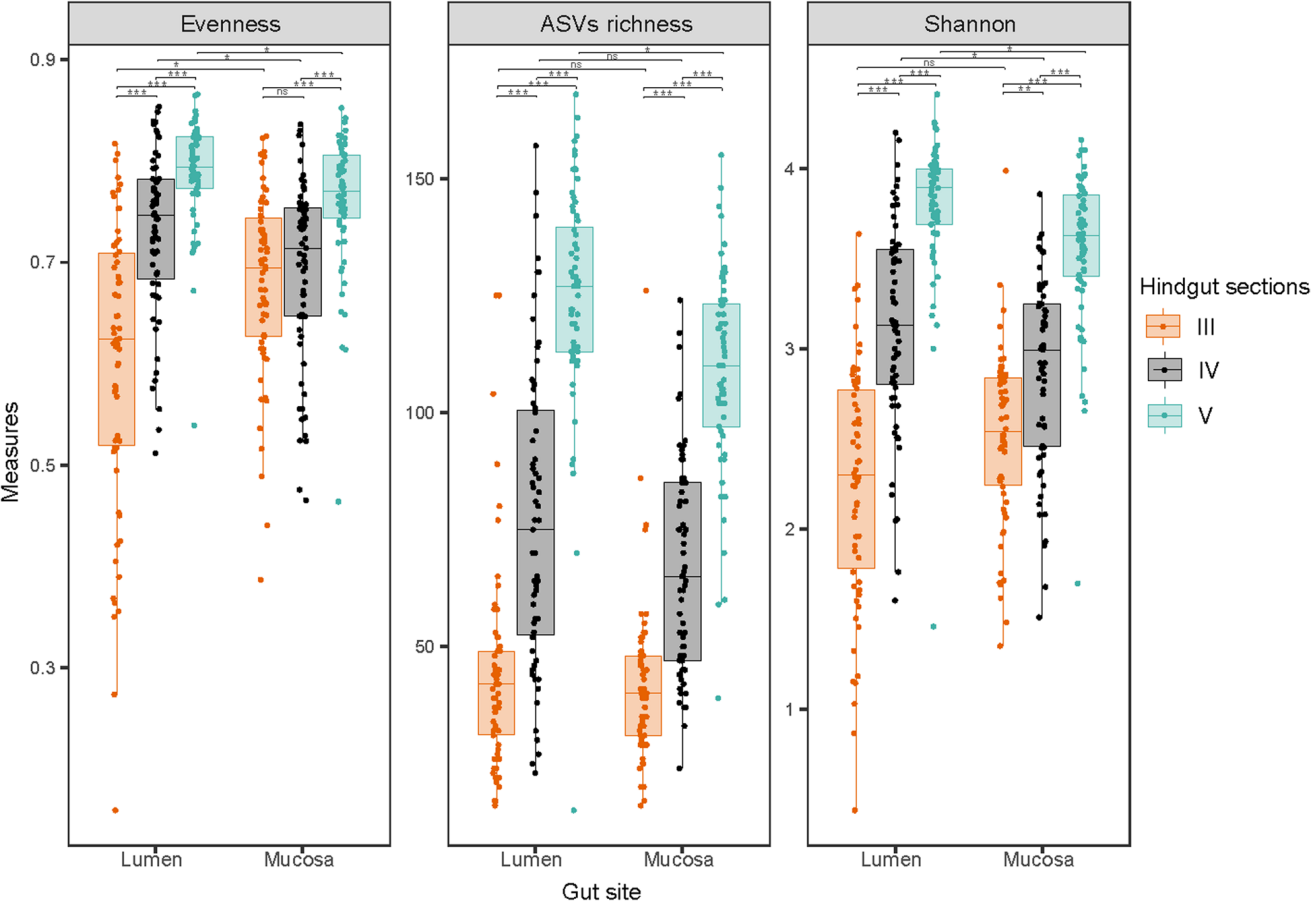
Boxplots of alpha diversity indices (Pielou’s evenness, ASV richness and Shannon) of gut sections III, IV and V in lumen and mucosa. Significance was tested with KW and a pairwise test using Dunn test on KW results

Alpha diversity indices (Pielou’s evenness, ASVs richness and Shannon) of gut sections III, IV and V in both lumen and mucosa from all sampling time points are shown in Fig. 4 and in Supplementary Fig. 6 (additional analysis for alpha diversity using Faith PD). Overall, all the diversity indices indicated that hindgut section III had the lowest diversity and hindgut section V the highest (Fig. 4 and Supplementary Fig. 6). Diversity increased significantly in the lumen (KW test, *p*-value < 0.001) and progressively from gut sections III to V (Fig. 4). Similar trends across gut sections were found for the mucosal samples in terms of ASV richness (KW test, *p*-value < 0.001) and Shannon index (KW test, *p*-value < 0.01 between section III and IV and *p*-value < 0.001 between gut section IV and V). Only the mucosal communities in sections III and IV exhibited similar evenness (Fig. 4 and Pielou’s index, *p*-value > 0.05). Lumen and mucosa communities of gut sections III and IV displayed similar ASV richness, however in section V diversity was significantly higher in the lumen (KW test, *p*-value < 0.05). Overall, results indicate that the highest diversity among all samples was in lumen section V (Fig. 4).

**Fig. 5:**
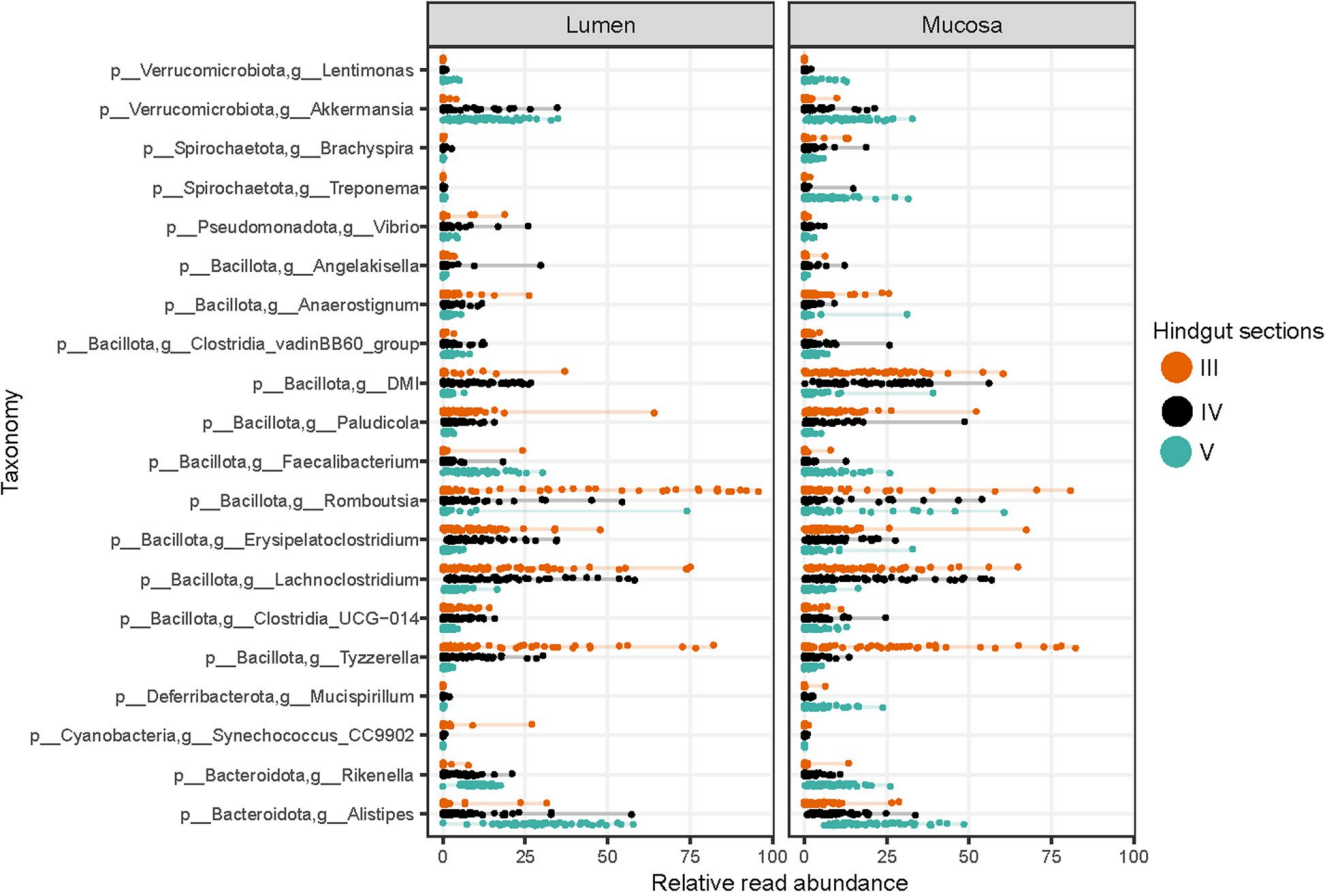
Genera most different in relative abundance between lumen and mucosa and/or hindgut sections. Genera were selected according to 1) *p*-value significance (***) based on the KW test (*p*-values were further adjusted with Benjamin-Hochberg, BH), and 2) having a relative abundance ≥ 10% in at least one gut section (III, IV and V) or gut site (lumen or mucosa)

We used a KW test (with BH adjusted *p*-values < 0.001) to identify and investigate the genera that contributed most to the differences in community structure found between lumen and mucosa and/or hindgut sections (Fig. 5). We identified twenty genera that were highly variable among our sampled communities, mainly from the phyla Bacillota and Bacteroidota. Those genera were either highly abundant in section III in both lumen and mucosa (i.e. genera belonging to phylum Bacillota such as *DMI*, *Rombutsia*, *Lachnoclostridium*, *Tyzzerella*, etc.) or were very abundant in section V (i.e. Verrucomicrobiota such as *Lentimonas* and *Akkermansia* and Bacteroidota including *Rikenella* and *Alistipes*). Alternatively, some genera differed in relative abundances between lumen and mucosa. For example, *Treponema* (phylum Spirochaetota) and *Mucispirillum* (phylum Deferribacterota) were very abundant in mucosa section V, but almost absent in the lumen (Fig. 5).

### Relative versus absolute abundance

**Fig. 6:**
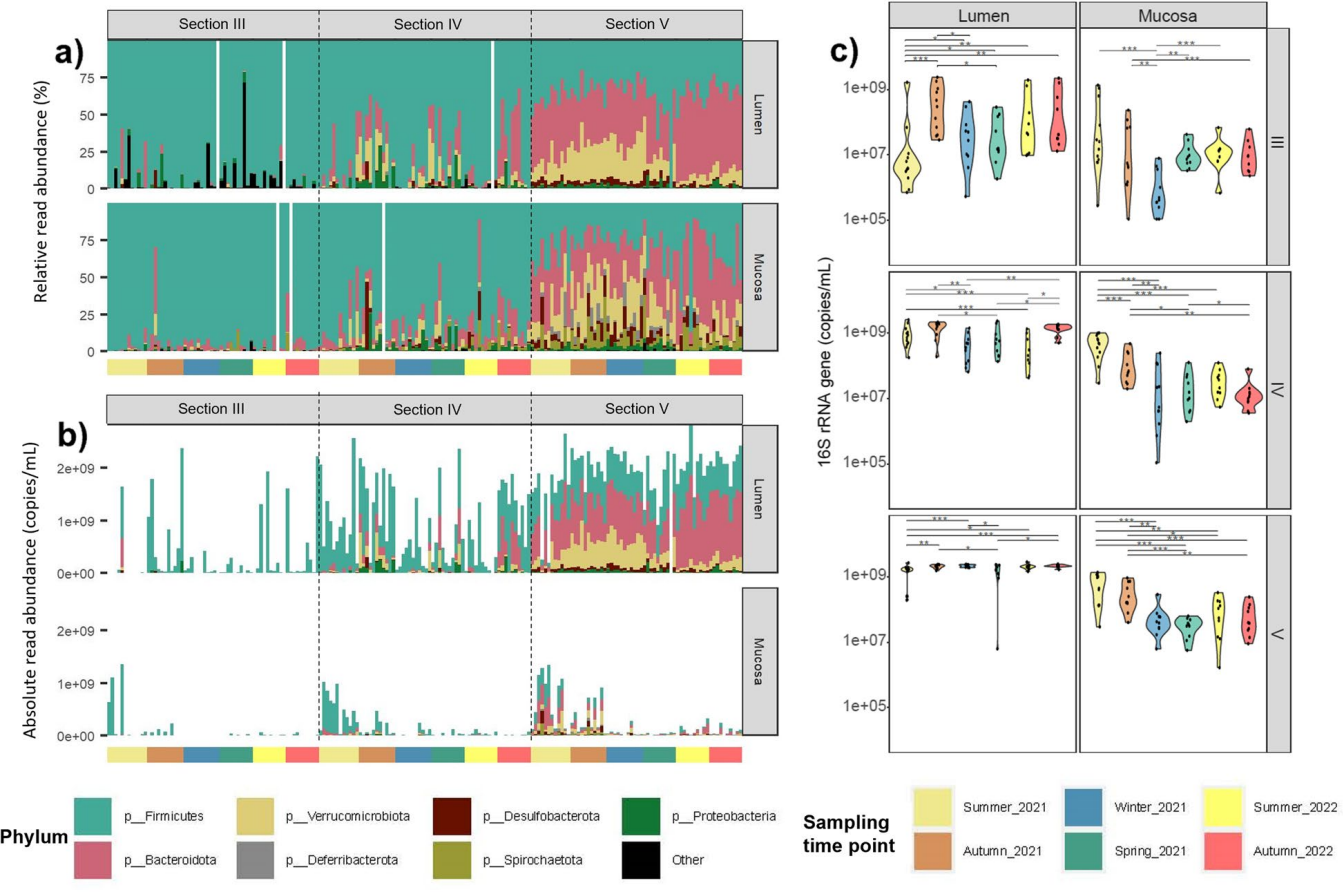
Microbial composition (%) and density (cp/mL) of *K. sydneyanus*. a) Relative (%) and b) absolute (copies/mL) abundance of individual samples of hindgut sections of *K. sydneyanus* in mucosa and lumen and across sampling time points. c) 16S rRNA gene (copies/mL) of hindgut sections of *K. sydneyanus* in mucosa and lumen and across sampling time points

Differences in community composition between gut sections remained relatively stable over time (i.e. among sampling time points), with little evidence of variation across (Fig. 1 and Supplementary Fig. 1). When comparing the phyla colonising the guts of individual fish we found that compositions were largely similar within each gut section (Fig. 6a). Nonetheless, variability among individual fish was apparent, especially in section III and IV of both lumen and mucosa. The community appeared more stable between individual fish in section V.

**Fig. 7:**
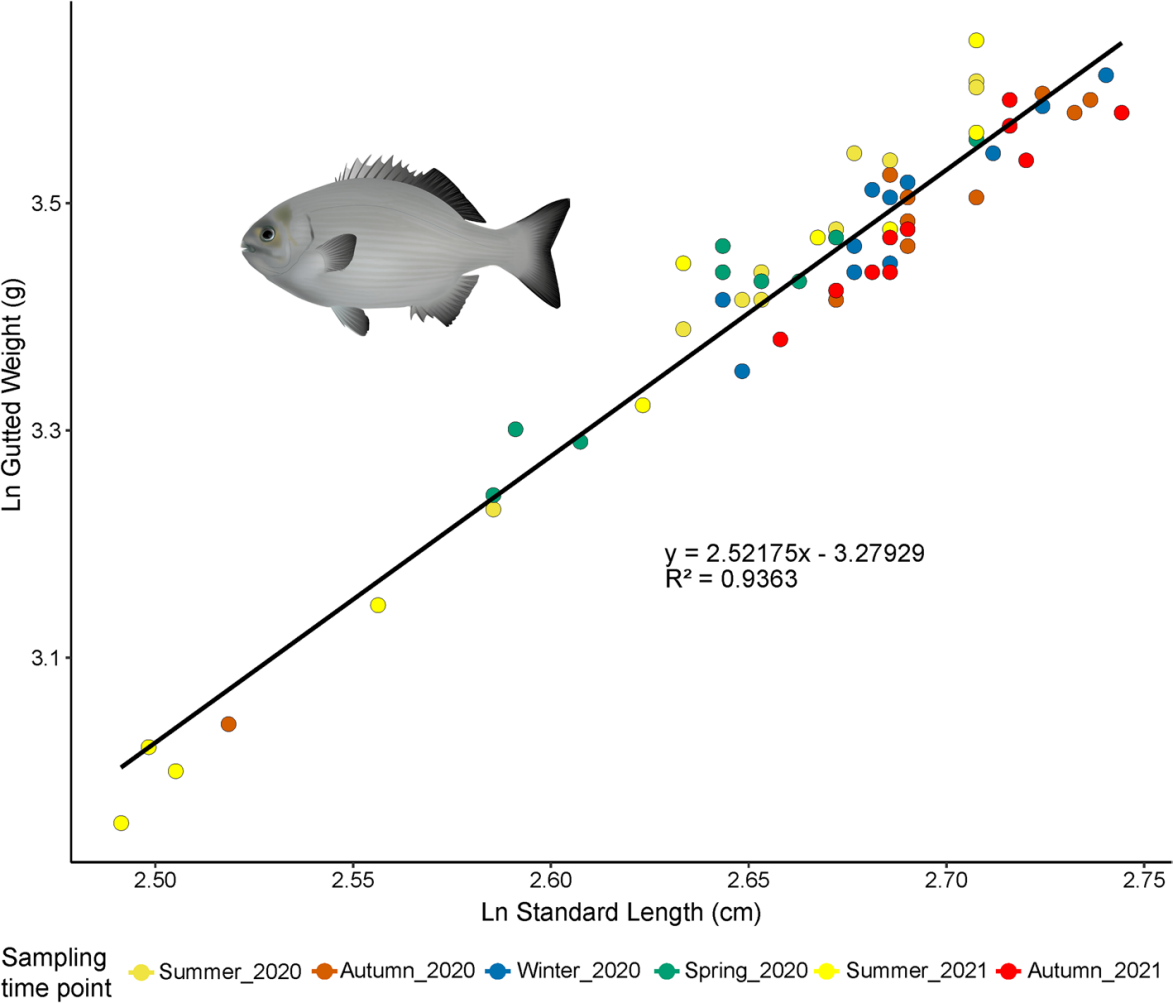
Weight and length relationship of *K. sydneyanus* collected across sampling time points. Weights is gutted following removal of the gut and other viscera. Ln is natural logarithm

To investigate the relationship between gut section, community composition and estimated absolute abundance we used ddPCR of the 16S rRNA gene to generate estimates of cell density (Fig. 6b and c). Estimated bacterial density was highly distinct among gut sections and variable among individual fish. Absolute abundance in the lumen was 2–3 × higher (≥ 2e + 09 copies/mL) than in mucosa (≤ 1e + 09 copies/mL), and increased from section III to section V (Fig. 6b). We found some variation in cell density (copies/mL) across sampling time points (Fig. 6c), however these differences appeared to be again due to individual or sporadic temporal variation (Fig. 6b), as there was no evidence of reproducible or consistent temporal patterns among time points. The considerable variation in ddPCR copy number in sections III and IV was not influenced by time of day as indicated by the linear correlation (r-squared *P*-value > 0.1, Supplementary Fig. 7), however section V density (copies/mL) decreased throughout the day as visually indicated by the linear model and negative slope (r-squared *P*-value < 0.01; Supplementary Fig. 7). Despite differences in microbial biomass among fish, data from all fish, collected across all sampling time points, fell on the same length–weight line (Fig. 7) indicating similar condition factors (values higher than 1 for all fish, Supplementary Fig. 8), suggesting that all the fish were in a similar good physiological condition.

### Co-occurrence of *Alistipes* and *Rikenella*

**Fig. 8:**
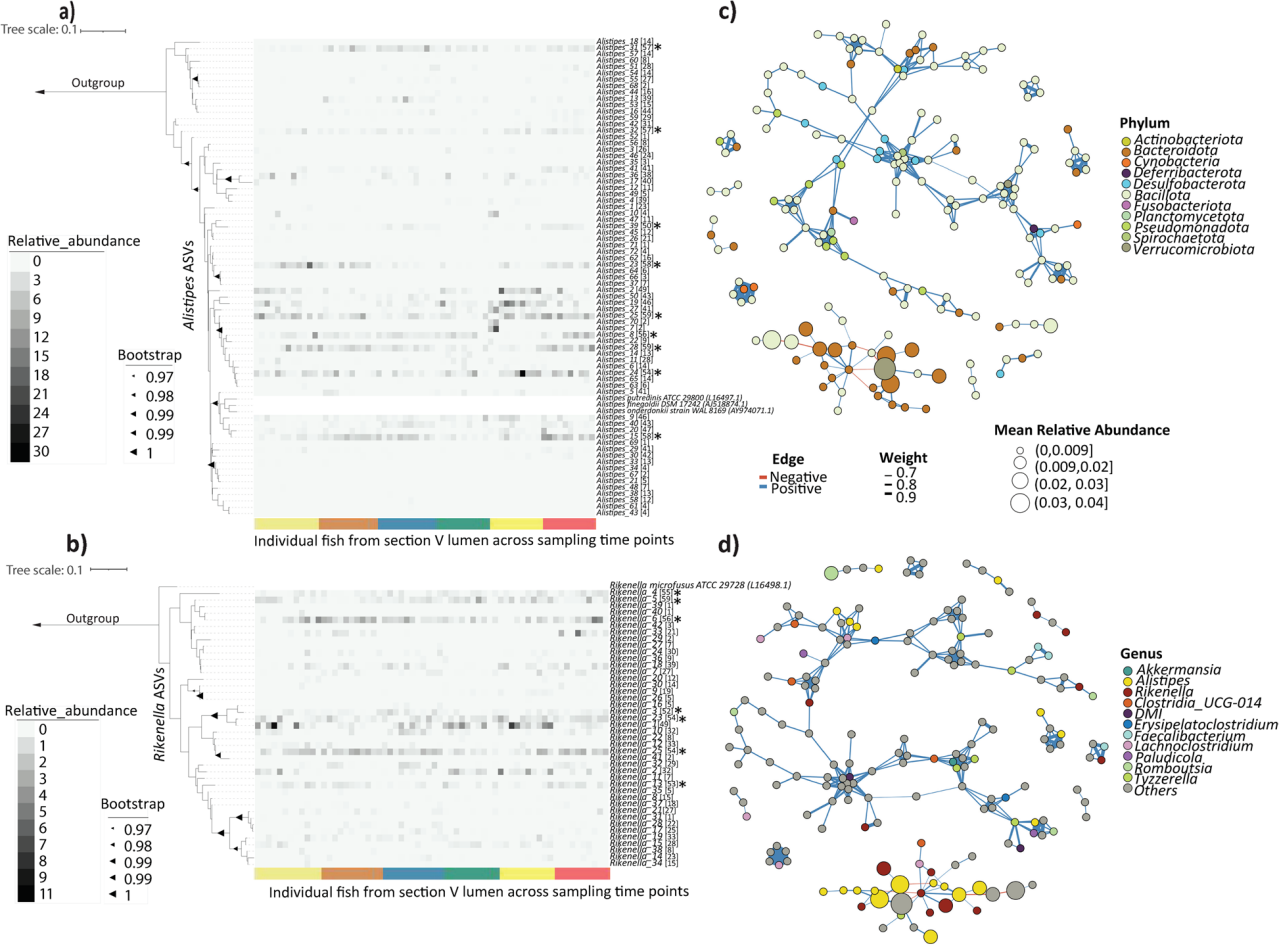
Co-occurrence of Bacteroidota. Phylogenetic trees of Bacteroidota ASVs for **a ***Alistipes* and **b ***Rikenella* recovered from lumen section V. The corresponding ASV relative abundances across samples is shown in adjoining heatmaps. Samples of lumen section V are individual fish ordered by sampling time points. Scale bar correpsonds to 0.1 substitutions per nucleotide position. 1000 boostrap. 8 (**c**) and (**d**) are network anlysis of lumen section V at all phylum and genus taxonomic level. Numbers in square bracket indicate the number of individiual fish for which one ASV is present. * refers to ASVs co-occuring in ≥ 50 individual fish

As members of the Bacteroidota were present at high abundance in section V (Fig. 6b), we evaluated its compositional variation between individual fish. To do this we built two phylogenetic trees with heatmaps of the most abundant Bacteroidota genera *Alistipes* and *Rikenella* (Fig. 8a and b) and examined bacterial co-occurrence at phylum and genus levels in lumen section V (Fig. 8d and b). Some ASVs of *Alistipes* and *Rikenella* co-occurred in almost all individual fish across multiple time points (e.g. *Alistipes 25, 8* and *24*, and *Rikenella 6, 23* and *25*; Fig. 8a and b). Other ASVs (i.e. *Alistipes 52*, *Alistipes 71*, *Alistipes 69* and *Rikenella 39*, *Rikenella 40*, *Rikenella 31*) were less abundant across fish/sampling time point (i.e. only present for one individual), and potentially individual-specific, or were too rare to consistently detect across multiple fish. Network analysis at the phylum level in lumen section V (Fig. 8c) revealed that the co-occurring phyla were mainly Bacillota and Bacteroidota. Bacillota showed positive edges, while Bacteroidota, the most abundant phylum, formed a cluster, made of both positive and negative edges (Fig. 8c). The negative and positive edges represent the interactions between bacterial taxa (Fig. 8c and d). This Bacteroidota cluster was formed by different *Alistipes* and *Rikenella*, indicating co-occurrence of these taxa in individual fish (Fig. 8d).

## Discussion

Recent studies have described a high degree of variability in gut microbiota composition of the temperate herbivorous fish *K. sydneyanus* between individual fish and hindgut sections [15, 17]. Here, we sought to investigate: (i) stability of microbial community composition across sampling time points (samples collected over six successive seasons in 2020 and 2021), hindgut sections (III, IV and V) and gut locations (lumen and mucosa); and (ii) processes influencing community assembly in the hindgut of *K. sydneyanus*.

### Temporal variation

Although we detected some statistically significant differences between sampling time points (Table 1, Fig. 3), which indicated the presence of some temporal variation, these changes could not be attributed to a seasonal effect, although further seasonal replication would be required to confirm this trend. No clear or similar patterns were found across the different temporal samples (i.e. replicate seasons: summer 2020 vs summer 2021 and autumn 2020 vs autumn 2021) collected at similar times of the year over two successive years indicating a lack of consistent temporal pattern (Figs. 3 and 6). Conversely, substantial variation in gene copy numbers (Fig. 6) were associated with diel collection time in section V, but not in sections III and IV (Supplementary Fig. 7). These patterns may be related to diel feeding activity and gut retention time. *K. sydneyanus* displays long gut retention times (~ 21 h in juveniles; [44]) and feeding activity is largely limited to crepuscular periods [48]. Overall, microbial community composition in *K. sydneyanus* appeared broadly stable over time, across gut sections, and gut sites (lumen and mucosa), as shown by taxonomic profiles (Fig. 1). Overall, the high variability in community composition among individual fish (Table 1 and Fig. 3) appears to be the cause of variability among different sample groups, including our time points (Figs. 2 and 6 of this study; [15, 17]). The length–weight relationship and condition factor of our sampled fish was consistent across all samples, indicating that all collected fish were in good condition (Fig. 7), ruling out some form of dysbiosis. A high degree of variability among individual fish has also been found in the skin microbiome [49]. In other words, any two samples may differ significantly simply due to inherent variation among individual fish.

In general, class *Clostridia* (phylum *Bacillota*) dominated gut sections III and IV of both lumen and mucosa and class *Bacteroidia* (phylum *Bacteroidota*) dominated section V (Figs. 1 and 5, Supplementary Fig. 1). *Bacillota* and *Bacteroidota* (formerly phyla *Firmicutes* and *Bacteroidetes*, respectively) are prevalent in the guts of many vertebrates, including humans, where they play an important role in individual health [50]. *Bacillota* and *Bacteroidota*, together with the phylum *Pseudomonadota* (formerly *Proteobacteria*) are abundant in the guts of herbivorous fishes such as surgeonfish [14, 24], rabbitfish [51, 52] and sea chubs [15–17, 21, 53]. These phyla (i.e. *Bacillota*, *Bacteroidota* and *Pseudomonadota*) are also characteristic of gastrointestinal tract of herbivorous vertebrates in general [10, 54, 55] and are associated with polysaccharide degradation and fermentation [10].

Temporal variation in gut communities in terrestrial herbivores is often associated with seasonal changes in diet or feeding behaviour [56, 57]. Seasonal and dietary variation in community composition was detected in the herbivorous marine European abalone *Haliotis tuberculata* despite the presence of a core microbiota that was established in juveniles and persisted in adults [58]. Diet in adult *K. sydneyanus* does not appear to vary seasonally, although an ontogenetic shift in diet from red and green algae in juveniles to brown algae in adults is associated with a shift in microbiota composition [23, 46]. However, this shift in community composition does not appear to be associated with the algal microbiomes themselves, as the microbiome of *Ecklonia radiata*, the main dietary alga of *K. sydneyanus*, differs substantially from that in the hindgut of the fish [15]. Similarly, in the Atlantic salmon (*Salmo salar*) the water environment was significantly different from the gut microbiota of the host fish, suggesting that the gut environment, in combination with diet, is central in determining microbial community assemblage [59].

### Variation associated with hindgut sections and locations (lumen versus mucosa)

Microbial community composition in the present study was broadly consistent with that reported elsewhere on the same host species [15, 17], showing variation between hindgut sections and individual fish. Microbial diversity increased from sections III to V and from mucosa to lumen, as reported previously [15, 17]. The increased diversity and density (Fig. 6b and c) along the gut of *K. sydneyanus* suggest that the majority of microbial taxa in the hindgut are autochthonous, and they are therefore unlikely to be facultative anaerobes [15].

In general, we found similar taxa between lumen and mucosa (Fig. 1), however, we detected some differences in relative read abundances of taxa, especially at the genus level (Fig. 5) and in absolute read abundance results (Fig. 6). Phylum level absolute read abundance was higher in the lumen than mucosa, particularly in lumen section V. Differences in lumen and mucosa gut microbiota are commonly reported [17, 60–62]. Lumen and mucosa provide distinct environments (i.e. different pH, oxygen level and antimicrobial peptides) and therefore can host different microbial populations [61]. One of the significant differences between lumen and mucosa in the present study involved the genera *Treponema* (Spirochateota) and *Mucispirillum* (Deferribacterota). These genera were present only in the mucosa, particularly in section V (Fig. 5). *Treponema* and *Mucispirillum* are usually considered pathogens in humans [63, 64], however *Mucispirillum* can be an antagonist to *Salmonella* in mice and thus protect against colitis [65]. *Treponema* can also serve as a mutualist in termites, where they contribute to nutrition via acetogenesis [66]. *Treponema* was also reported in the mucus of killer whales (*Orcinus orca*) [67] and in fecal samples of other species of whales (e.g. *Eubalaena glacialis* and *Megaptera novaeangliae*) [68]. However, the function of *Treponema* in marine animals remains unclear [67].

### Microbiota community assembly

Community assembly can involve multiple factors, including the processes of environmental selection, historical contingency, random sampling/neutral effects and dispersal limitation [5, 69]. These processes and the factors influencing gut microbiota variation (i.e. season, gut location, host individuals, fish ontogeny, etc.) are essential to understanding how microbial community assembly occurs in the gut of *K. sydneyanus.* Community assembly in these fish is of interest since the gut microbiota are critical to fish nutrition [70].

The absence of bacteria detected from molecular and microscopic analysis of lionfish eggs indicate that in broadcast-spawning fish the gut-associated microbiota are likely acquired from the environment rather than being vertically transmitted [29]. Similarly, the apparent lack of vertical transmission in *K. sydneyanus* [17, 23] suggests that gut microbial community assembly is dependent on the surrounding environment in which the fish live, and perhaps associations with conspecifics.

High variation among individual fish in microbiota community composition and temporal stability across sampling time points suggest that initial community assembly involves a mixture of environmental selection and random sampling/neutral effects. The large variation in the taxonomic composition of gut communities in *K. sydneyanus* suggests that there is a large environmental pool of potential community members that can colonise the gut. Which taxa colonise a particular fish might be due to a mixture of random variation, dispersal limitation and historical contingency, i.e. factors influencing which taxa or combination of taxa are present at a particular host location at a given time. Macroalgal dietary treatments (*Ulva rigida* or *Ascophyllum nodosum*) used to determine the drivers of microbial community assembly suggested that dispersal limitation was a fundamental process in community assembly in the hindgut of Atlantic Cod [71]. However, historical contingency may also play a role in community assembly in *K. sydneyanus*, it is likely once taxa become established they appear to be resilient to change. The gut environment also influences community assembly and this filtering can decrease with host ontogenetic development [72]. The dynamics of substrate depletion along the gut of *K. sydneyanus* following feeding, i.e. the depletion of storage metabolites in the proximal intestine, may limit the impact of seasonal changes in dietary algae. Taxa that were first to colonise the gut of Atlantic Cod shaped the environment for subsequent species that became constituent members of the community (i.e. some *Bacteroidetes* and *Firmicutes* species) [71]. Similarly, the co-occurrence of some Bacteroidota groups across *K. sydneyanus* individuals (Fig. 8c and d) could indicate that these taxa are established members of the gut microbiome of *K. sydneyanus*, i.e. early colonizing members with a selective advantage in the host gut.

*Alistipes* and *Rikenella* represented the dominant genera in Bacteroidota lumen section V (Figs. 5 and 6). Some strains of these genera co-occurred in all samples (Fig. 8), suggesting that these bacteria may provide functions that are essential for the host. In mammals, the abundance of *Rikenella* and *Alistipes* in the gut microbiota is positively associated and positively correlated with the production of SCFA [73, 74]. Positive interactions may suggest commensalism and protocooperation, while negative interactions may indicate competition among microbiota [75]. The co-occurrence of Bacteroidota (*Rikenella* and *Alistipes)* in the *K. sydneyanus* microbiome involves both positive and negative interactions (positive and negative edges in Fig. 8c and d), suggesting the possibility that environmental selection and historical contingency may play an important role in how those strains of bacteria co-operate and/or compete for resources to become established members of the gut microbiome. Community composition may be shaped by particular keystone or other taxa performing critical functions (i.e. perhaps Bacteroidota in our study, which likely provide nutritional service to the host and influence the gut microbiota), and the loss or gain of these species may influence the network of interactions that drive community assembly processes and thus microbiota composition [6]. Another study suggests that herbivorous diet favours co-occurring consortia of bacteria containing keystone species [76]. Co-occurrence network analysis in other environments (both free-living and human-associated microbiota) shows that phylogenetic relatedness and functional associations among bacteria were stronger predictors of coexistence than phylogenetically distant bacteria [6, 77]. This agrees with our results where the co-occurring clusters of bacteria that we found in the Bacteroidia network plot (Fig. 8d) were very phylogenetically similar (i.e. the Bacteroidota *Alistipes* and *Rikenella*).

## Conclusions

This is the first detailed characterization of the gut microbiota of *K. sydneyanus* that investigated hindgut sections and locations across sampling time points using both relative (%) and estimated absolute (copies/mL) read abundances. Although we detected some statistically significant differences between sampling time points, indicating the presence of temporal variation, these changes could not be attributed to seasonal factors (i.e. sampling time points of the same kind such as summer 2020 and summer 2021, autumn 2020 and autumn 2021 showed different trends), but instead reflect high variability among the gut microbiota of host individuals. The present study and others [15–17] highlight the strong variability among individual fish of the family Kyphosidae. Lack of evidence for clear temporal trends in the taxonomic profiles of gut microbiota, combined with high variation among host individuals, suggests that initial community assembly could involve environmental selection and random sampling/neutral effects. However, historical contingency and dispersal limitation may also influence the dynamics of community assembly in fish [71]. In this study, ddPCR results provide new information on estimated bacterial density, showing that bacteria, in particular Bacteroidota, had the highest estimated density (copies/mL) in distal-most lumen section V, where SCFA concentrations are highest. Bacteroidota genera *Alistipes* and *Rikenella* may play important roles in the breakdown of seaweed into useful compounds for the fish host. It is likely that both Bacteroidota are also early colonizer and established members of the fish microbial communities. It can be challenging to identify the processes that influence the community assembly in the gut of wild caught herbivorous fish, due to the many stochastic factors and/or processes occurring and/or interacting in nature. One approach to further determining the processes driving community assembly, and confirming whether Bacteroidota are early colonisers, could be to compare these data with juvenile individuals of *K. sydneyanus* collected from the same geographic location, and to perform a meta- analysis of these data and the data available from other *Kyphosus* species. A comparison with juvenile individuals would also provide insights into the gut microbiota of *K. sydneyanus* when fish undergo a dietary change from red and green algae (consumed by juveniles) to brown seaweed (consumed by adults).

## Material and methods

### Sample collection

Ten to 12 adult specimens of *K. sydneyanus* were collected for time points in 2020 (all four seasons) and for the summer and autumn of 2021. Winter and spring sampling in 2021 were not possible due to COVID-19 limiting our seasonal sampling to six collections. Therefore, seasons are only considered as sampling time points throughout the manuscript. by spear gun on snorkel near Great Barrier Island, Hauraki Gulf, New Zealand (36˚17ʹ S, 175˚20ʹ E). Water temperature was recorded for each season (Supplementary Table 2). Fish standard length, weight and collection time were recorded for each specimen (Supplementary Table 2). Lumen samples of intestinal content, including microbiome, were collected from gut sections III, IV and V following Mountfort et al. (2002) [36]. Following collection of lumen content, the three intestine sections were slit longitudinally, the gut wall was thoroughly rinsed in autoclaved Milli-Q water to remove gut contents and gut fluid and then scraped with a spatula to sample the mucosal flora following Stevenson et al. (2022) [17]. This yielded six samples per individual fish, giving a total of 384 samples overall. The lumen gut content and mucosa samples were immediately frozen in liquid nitrogen and transported to the University of Auckland, New Zealand for further analysis.

### DNA extraction, PCR amplification and sequencing

DNA was extracted from samples of lumen content and mucosa from hindgut sections III, IV and V (approximately 250 mg of material) following the manufacturer’s instructions for the DNeasy PowerSoil DNA Isolation Kit (Qiagen, Hilden, Germany). DNA quality and quantity were checked via NanoPhotometer (Implen, Germany) and Quibit 3.0 fluorometric quantitation (Thermo Fisher Scientific, Waltham, MA, USA). 16S rRNA gene amplifications of the V3-V4 region were performed in single reactions per sample using 0.75 μl (10 μM stock solution) and 0.5 μl (20 mg/ml) of bovine serum albumin (BSA) with the tagged and barcoded primers 341F (5'-CCTACGGGNGGCWGCAG-3') and 785R (5'-GACTACHVGGGTATCTAATCC-3') [78, 79]. Reactions were carried out using 12.5 μl of 2 × KAPA HiFi HotStart (KAPA Biosystems, Woburn, MA, USA). Volume of genomic DNA (1–2 μl) and ultrapure molecular biology water were adjusted according to DNA concentration. PCR reactions (25 μl) were run with negative controls.

Thermocycling conditions consisted of 3 min at 95 °C for the initial denaturation step, followed by 25 cycles of 95 °C for 30 s, 55 °C for 30 s, 72 °C for 30 s and a final extension of 5 min at 72 °C. Samples were purified using Agencourt AMPure XP magnetic beads (Beckman Coulter, CA USA) and quality checked with a Qubit fluorometer. Library preparation and 2 × 300 bp paired end sequencing was performed using the Illumina MiSeq platform with V3 chemistry at Auckland Genomics (University of Auckland, NZ).

### Amplicon data analysis

Amplicon sequences were processed using QIIME2 (v 2021.2) [80]. Demultiplexed amplicon sequences were read-joined, quality filtered (Q score cutoff of 25) and denoised (with singletons removed) in order to generate an ASV table [80–82]. Poor quality samples (samples G294IIIL and G292IIIM) were discarded. Taxonomy of ASVs was assigned using SILVA database v138 [83–85]. Finally, rarefaction was carried out at 1052 sequences (Supplementary Fig. 9) per sample using QIIME2 [80], leaving a total of 1720 ASVs across samples. After subsampling, samples with a low number of reads (< 1052) were removed from the dataset (i.e. G245IVM, G296IIIM and G293IVL) leaving a total of 378 samples out of 384. Data were visualized using R v4.0.1 with RStudio software version 1.2.1335 and the R package ggplot2 v3.3.5 [86]. Statistical analysis was performed using permutational multivariate analysis of variance (PERMANOVA) with the function adonis2 of vegan v2.5–7 and Kruskal Wallis with post-hoc Dunn test using the pacakge dunn.test v1.3.5 [86]. We performed statistical analysis with variables based on gut sections, gut locations (lumen and mucosa) and six conseguitive sampling time points (summer 2020, autumn 2020, winter 2020, spring 2020, summer 2021, autumn 2021). Structure of RDA analysis was performed using both categorical and quantitative environmenal variables, as performed in studies with similar dataset structure [87, 88].

### Droplet digital PCR (ddPCR) for 16S rRNA gene quantification

The abundance of the bacterial communities was estimated using droplet digital PCR (ddPCR). Each reaction was run with 11 μl of 2X QX299 ddPCR EvaGreen supermix (Bio-Rad Laboratories, CA, US), 7.4 μl of DNase-free water (ultrapure water from Thermo Fisher Scientific) and 2 μl of DNA sample for a final volume of 22 μl and 0.8 μl of 350 nm of forward (341F) and reverse (785R) primers [79]. The primers were the same as used for the 16S rRNA gene PCR amplifications but without the adapters. As ddPCR method uses 16S primers which can sometimes also amplify 12S regions of mitochondria we checked for their amplification in our ASV table. On average mitochondria only represent 0.4% of the microbiota. Moreover, we emphasize here and throughout the manuscript that ddPCR results of 16S amplicons represent estimated value.

In order to optimize the quantification of bacterial communities using degenerate 16S amplicon primers (wide spectrum/non-species-specific) different conditions were tested including serial dilutions of DNA template (10, 100, 500, 1000, 10,000), presence or absence of restriction enzymes in the mix, temperature gradients (52℃ to 60℃) and different primer concentrations (250 nm, 350 nm, 500 nm). The optimized ddPCR protocol consisted of: (i) 1/500 sample dilutions; (ii) an annealing temperature of 55℃; (iii) primer concentration of 350 nm; and (iv) thresholding performed manually to the negative control. To check for samples and background contamination three negative controls (no template) were included in each 96-well plate and the average of those negatives was deducted from the samples.

Analysis of the samples was performed with QX manager 1.2 to generate number of copies per μl. To calculate the copies/ml of samples ddPCR results were multiplied × 11 (ddPCR reaction volume), × 500 (sample dilution), × 50 (original DNA elution volume) and divided by 250 (mg of samples used for DNA extraction). The final result was multiped by 1000 to convert copies/μl into copies/ml. The resulting values of bacterial density were multiplied by the relative read abundance from 16S rRNA gene amplicon sequencing to generate the estimated absolute abundance.

### Phylogeny, relative abundance and network analysis of *Alistipes* and *Rikenella*

Two phylogenetic trees and corresponding relative abundance heatmaps were constructed using Bacteroidota ASV sequences. The dataset included 72 *Alistipes* ASV sequences recovered from the present study and three *Alistipes* 16S full length reference sequences from GenBank (https://www.ncbi.nlm.nih.gov/genbank/). A gammaproteobacterium, *Marinagarivorans algicola*, was used as an outgroup to root the *Alistipes* phylogenetic tree. The tree was merged with a heatmap of the corresponding ASV relative abundances generated from this study. The *Rikenella* phylogenetic tree and heatmap used 42 *Rikenella* ASVs from the present study and one 16S V3-V4 full length *Rikenella* reference sequence from GenBank. Sequences were aligned with MUSCLE v3.8.31 to generate a multiple sequence alignment and trimmed with trimAl v1.4.1-Gc-9.2.0 to remove poorly aligned regions from the alignment and increase the quality of the subsequent analysis. A maximum-likelihood phylogenetic tree was constructed using FastTree v2.10-gimkl-2018b [89, 90]. Finally, the trees were visualized in iTOL [91]. Co-occurrence network analyses for lumen section V at phylum and genus level were performed using the Phylosmith R package (phylosmith v1.0.6 https://schuyler-smith.github.io/phylosmith/ [92]).

### Supplementary Information

**Additional file 1:** **Supplementary Figure 1.** Bacillota and Bacteroidota relative abundance across sampling time points and gut sections in lumen. **Supplementary Figure 2. **Redundancy discriminant analysis (RDA) of ASVs coloured by Phylum level. **Supplementary Figure 3. **Microbiome Multivariable Association with Linear Models (MaAsLin2) used to test significant multivariable associations. **Supplementary Figure 4. **Weighted UniFrac PCoA of lumen and mucosa gut sites showing sampling time point within each gut section (III, IV and V). **Supplementary Figure 5. **Redundancy discriminant analysis (RDA) of samples. **Supplementary Table 1. **Table showing analysis of variance (ANOVA) for Redundancy discriminant analysis (RDA) shown in Supplementary Figures 2 and 5. **Supplementary Figure 6. **Faith phylogenetic diversity (Faith PD) analysis for alpha diversity for both lumen and mucosa sites of hindgut section III, IV and V. **Supplementary Figure 7.** Microbial densities at each fish collection time point. Densities are based on ddPCR of 16S rRNA genes (copies/mL) in samples from lumen sections III, IV and V. **Supplementary Figure 8. **Condition factor of individual fish coloured by sampling time points. **Supplementary Figure 9. **Rarefaction sequencing curves. **Supplementary Table 2. **Collection details for *K. sydneyanus. *GBI indicates Great Barrier Island and LBI indicates Little Barrier Island.

## Data Availability

The raw reads analysed in the current study have been deposited at the National Center For Biotechnology Information (NCBI, https://www.ncbi.nlm.nih.gov/) and are available under the BioProject ID: PRJNA930464 or through the following link https://www.ncbi.nlm.nih.gov/bioproject/PRJNA930464.
