# Supplementary material for: Host individual and gut location are more important in gut microbiota community composition than temporal variation in the marine herbivorous fish Kyphosus sydneyanus
Source: BMC Microbiol. 2023 Sep 29;23:275. doi: 10.1186/s12866-023-03025-2 (PMC10540440; doi:10.1186/s12866-023-03025-2)
Supplement: Supplementary file 1 — Additional file 1: Supplementary Figure 1. Bacillota and Bacteroidota relative abundance across sampling time points and gut sections in lumen. Supplementary Figure 2. Redundancy discriminant analysis (RDA) of ASVs coloured by Phylum level. Supplementary Figure 3. Microbiome Multivariable Association with Linear Models (MaAsLin2) used to test significant multivariable associations. Supplementary Figure 4. Weighted UniFrac PCoA of lumen and mucosa gut sites showing sampling time point within each gut section (III, IV and V). Supplementary Figure 5. Redundancy discriminant analysis (RDA) of samples. Supplementary Table 1. Table showing analysis of variance (ANOVA) for Redundancy discriminant analysis (RDA) shown in Supplementary Figures 2 and 5. Supplementary Figure 6. Faith phylogenetic diversity (Faith PD) analysis for alpha diversity for both lumen and mucosa sites of hindgut section III, IV and V. Supplementary Figure 7. Microbial densities at each fish collection time point. Densities are based on ddPCR of 16S rRNA genes (copies/mL) in samples from lumen sections III, IV and V. Supplementary Figure 8. Condition factor of individual fish coloured by sampling time points. Supplementary Figure 9. Rarefaction sequencing curves. Supplementary Table 2. Collection details for K. sydneyanus. GBI indicates Great Barrier Island and LBI indicates Little Barrier Island. [file 12866_2023_3025_MOESM1_ESM.docx]

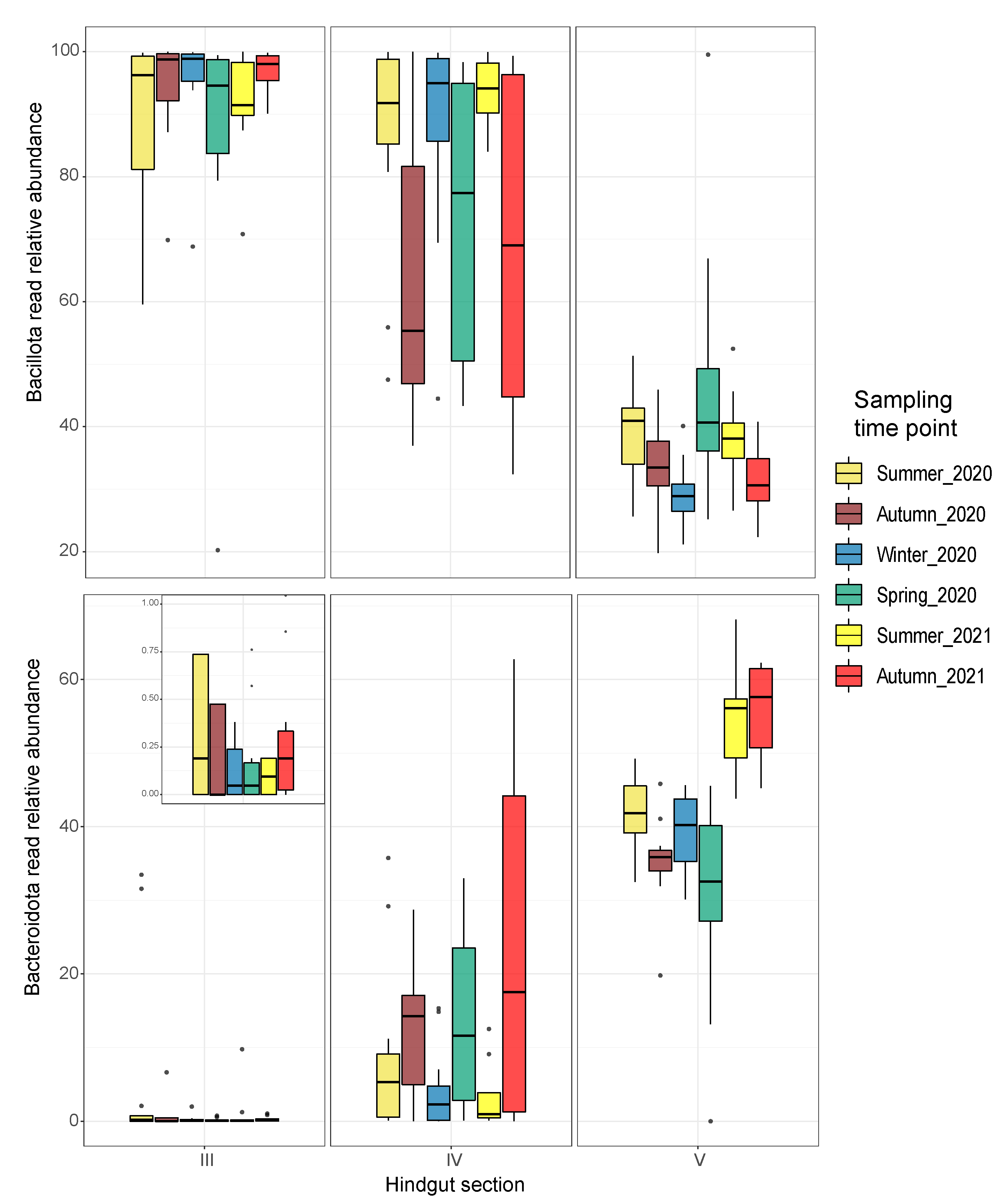


**Supplementary Figure 1**. Bacillota and Bacteroidota relative abundance across sampling time points and gut sections in lumen.


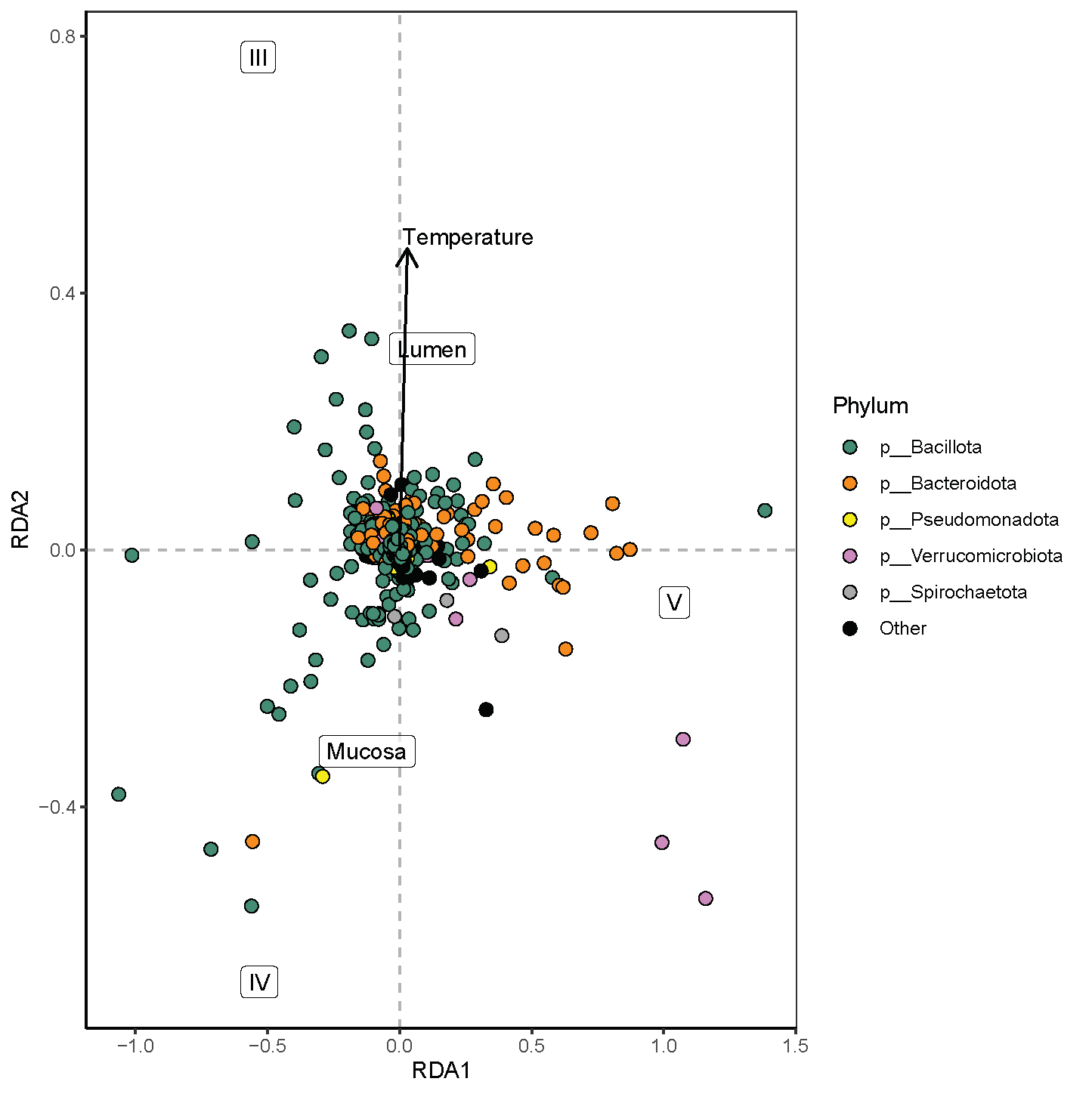


**Supplementary Figure 2.** Redundancy discriminant analysis (RDA) of ASVs coloured by Phylum level. The environmental factors considered were the categorical variables of gut locations (mucosa/lumen) and gut sections (III, IV, V), and the quantitative variable of temperature associated with sampling time points. ASV table was transformed using “rclr” method and scaling was performed using “species” method through the vegan package in R studio.


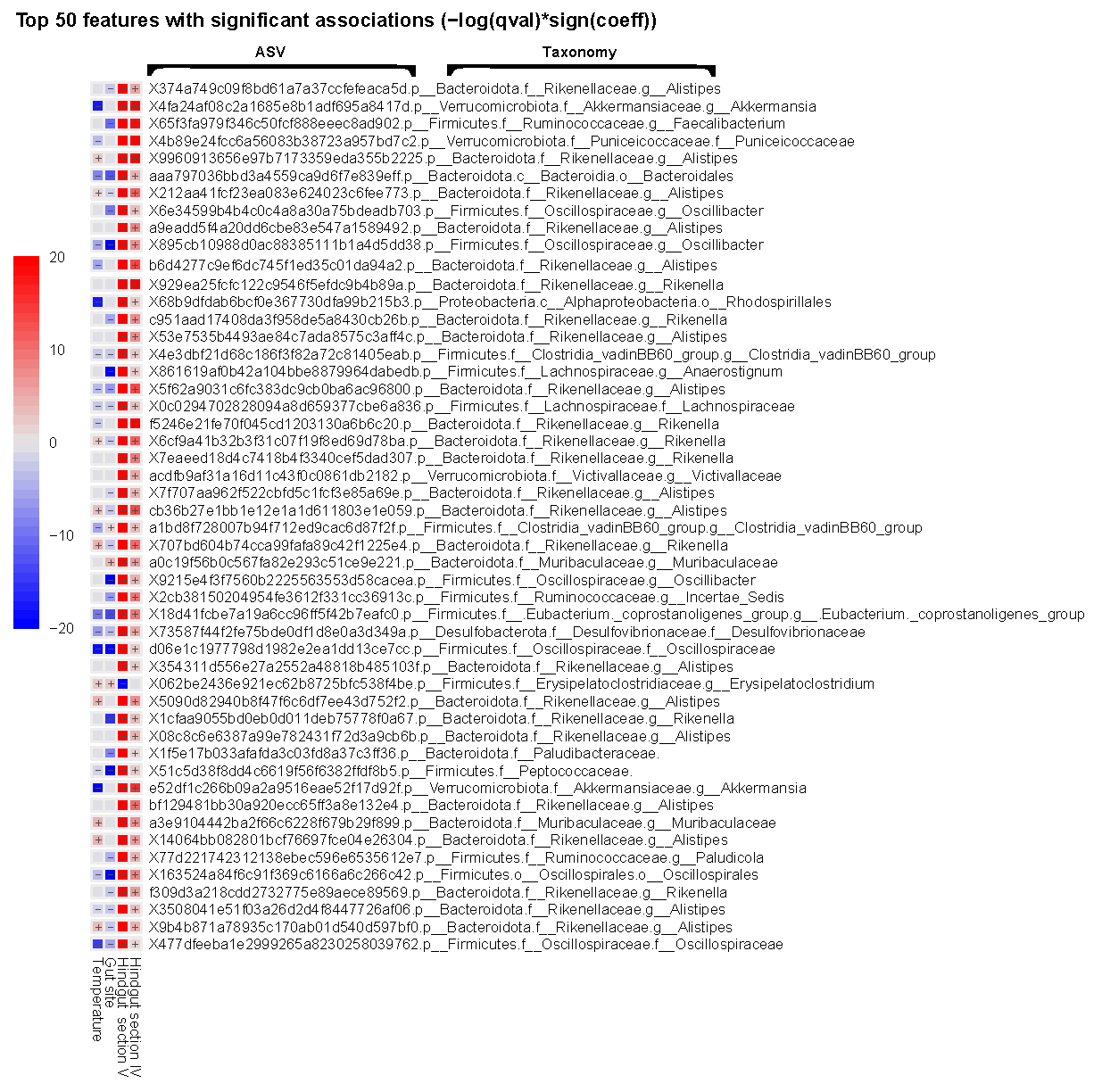


**Supplementary Figure 3.** Microbiome Multivariable Association with Linear Models (MaAsLin2) used to test significant multivariable associations. Fixed effects used for the analysis were temperature, gut sections, and gut sites. Reference variables for the categorical factors were lumen for gut sites and section III for hindgut sections.


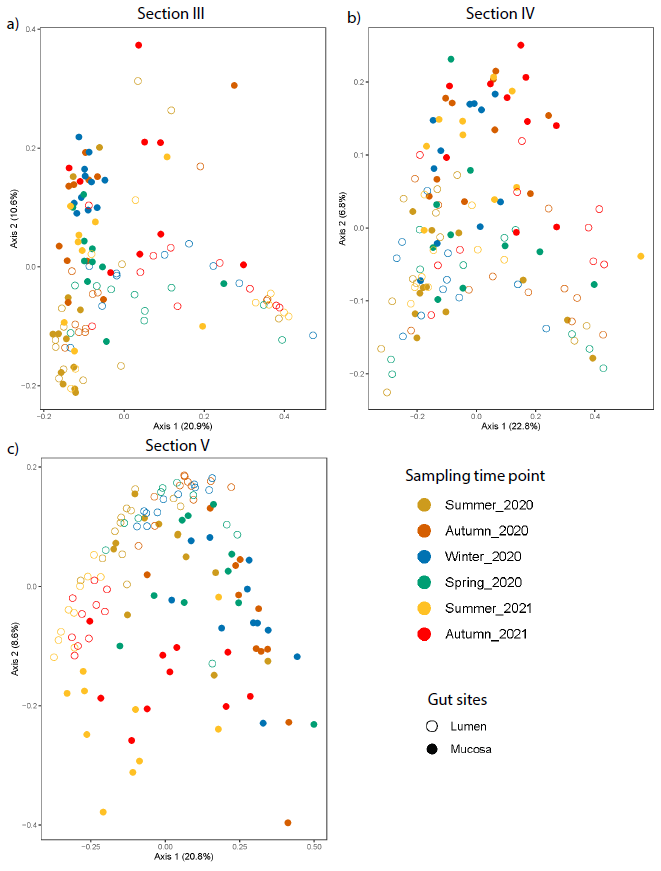


**Supplementary Figure 4.** Weighted UniFrac PCoA of lumen and mucosa gut sites showing sampling time point within each gut section (III, IV and V). a) is gut section III, b) is gut section IV and c) is gut section V.


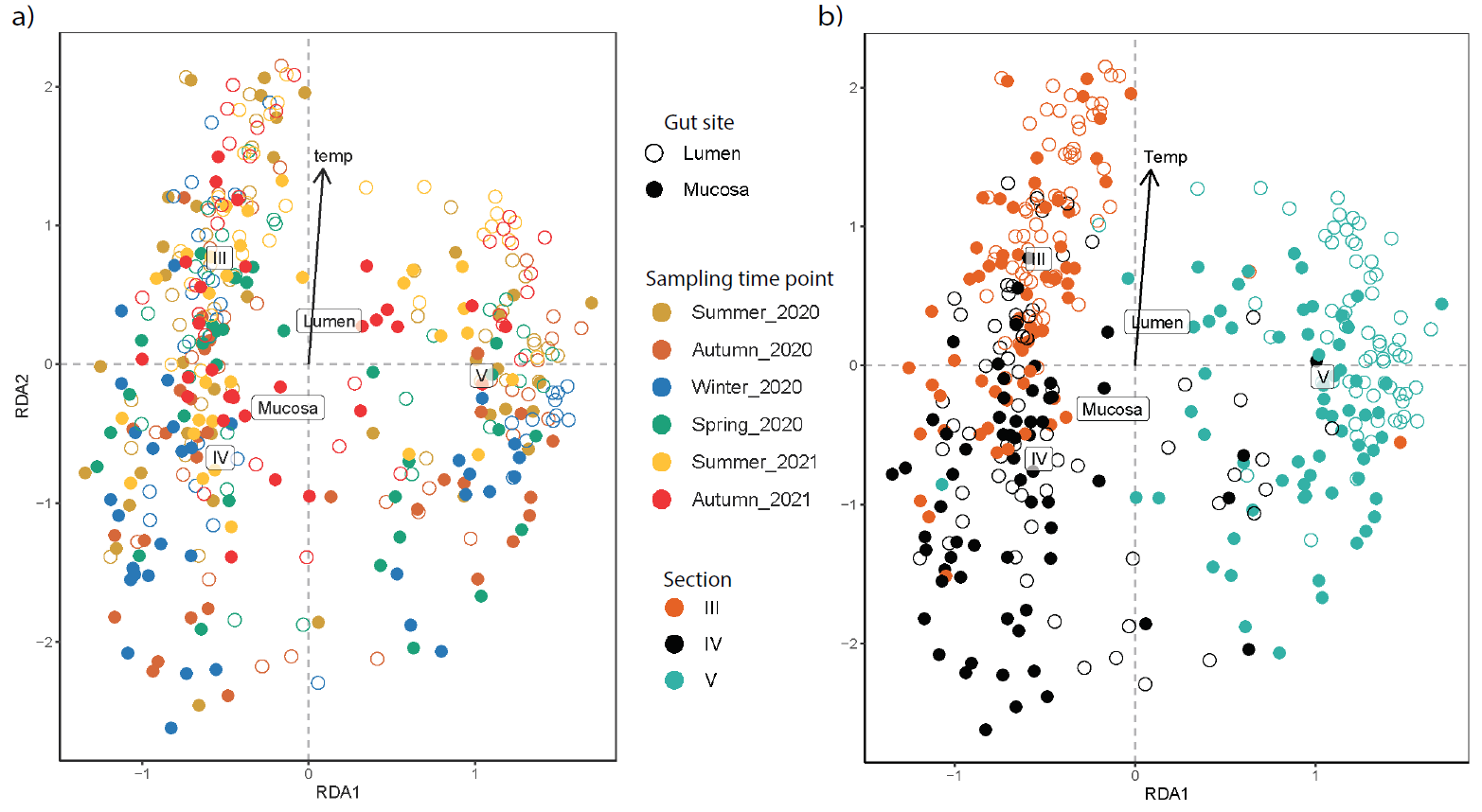


**Supplementary Figure 5.** Redundancy discriminant analysis (RDA) of samples. a) Samples coloured by sampling time points, b) samples coloured by gut sections. The environmental factors considered were the categorical variables, gut location (mucosa/lumen) and gut section (III, IV, V), and the quantitative variable, seasonal temperature. ASV table was transformed using “rclr” method and scaling was performed using “species” method through the vegan package in R studio.

**Supplementary Table 1.** Table showing analysis of variance (ANOVA) for Redundancy discriminant analysis (RDA) shown in Supplementary Figures 2 and 5.

| **Variables** | **Df** | **Variance** | **F** | **Pr(>F)** |
| --- | --- | --- | --- | --- |
| Temperature | 1 | 1.394 | 4.8449 | 0.001*** |
| Gut sites | 1 | 1.720 | 5.9766 | 0.001*** |
| Gut sections | 2 | 13.010 | 22.6070 | 0.001*** |
| Residual | 373 | 107.327 |  |  |


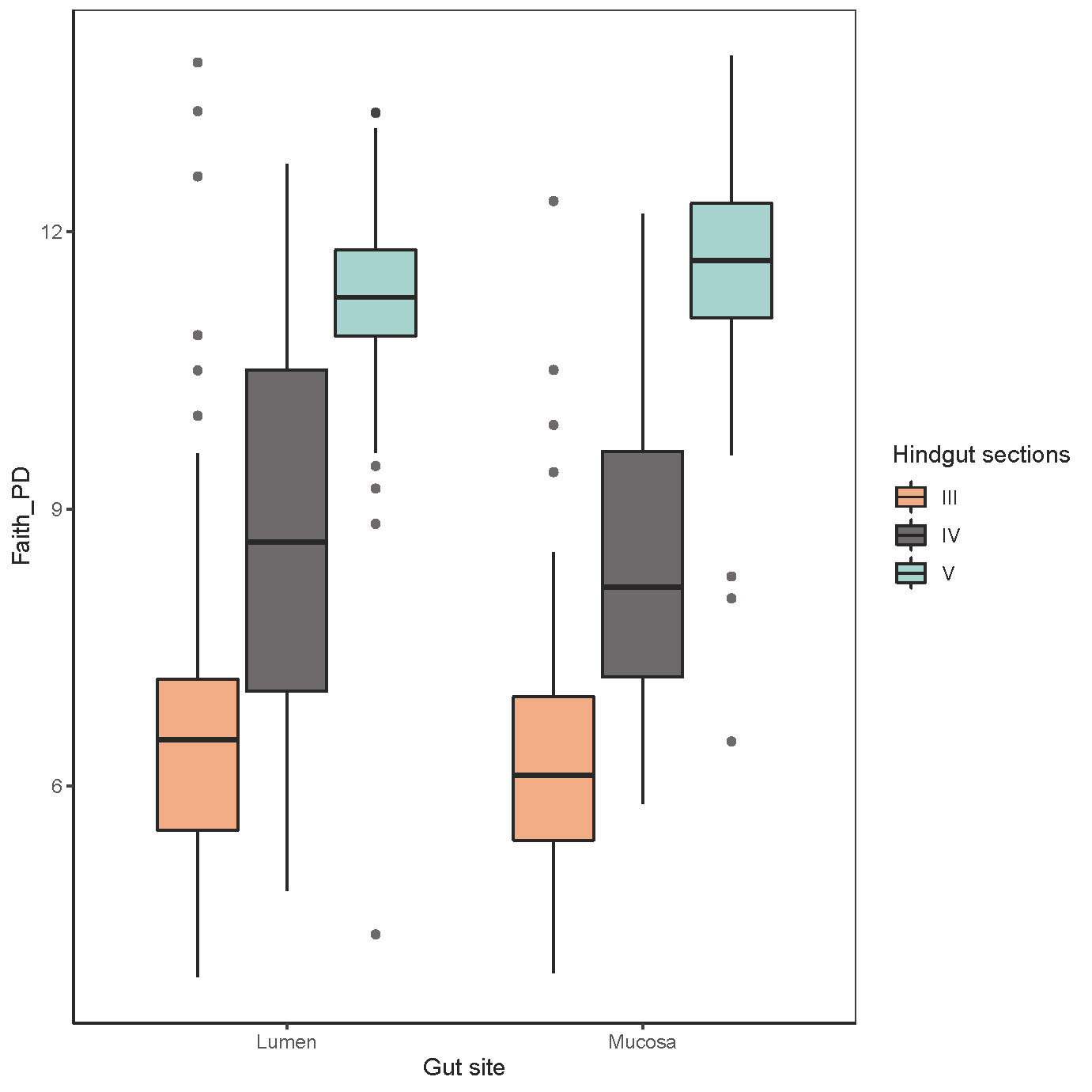


**Supplementary Figure 6.** Faith phylogenetic diversity (Faith PD) analysis for alpha diversity for both lumen and mucosa sites of hindgut section III, IV and V.


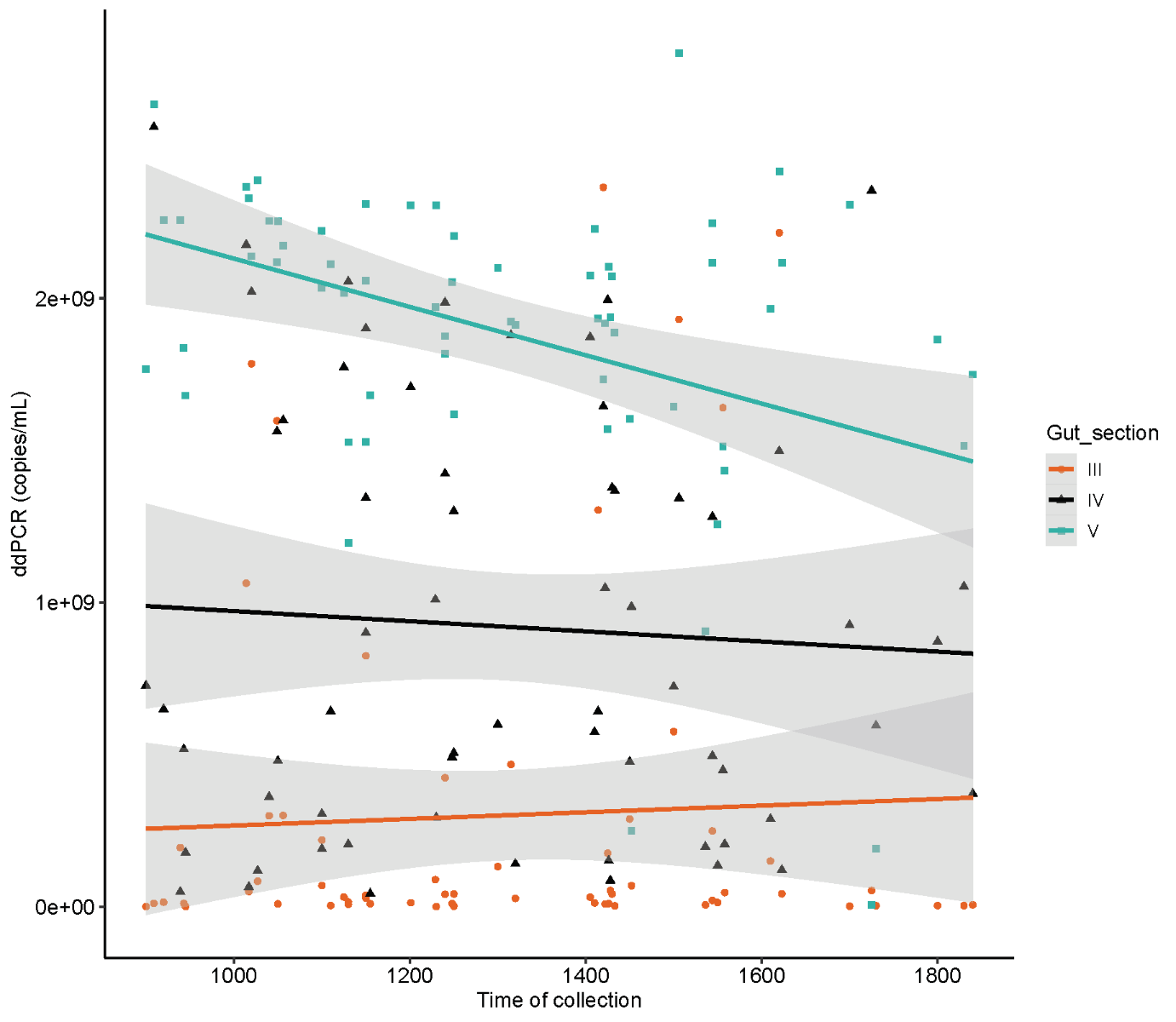


**Supplementary Figure 7.** Microbial densities at each fish collection time point. Densities are based on ddPCR of 16S rRNA genes (copies/mL) in samples from lumen sections III, IV and V. Regression line equation for section III is y= 108994x + 158093228 with R^2^= 0.00221 and P> 0.1; section IV is y = -166462x +1138379907 with R^2^= 0.003629 and P>0.1; section V is y= -794298x + 2925104522 with R^2^ = 0.1509 and P <0.01


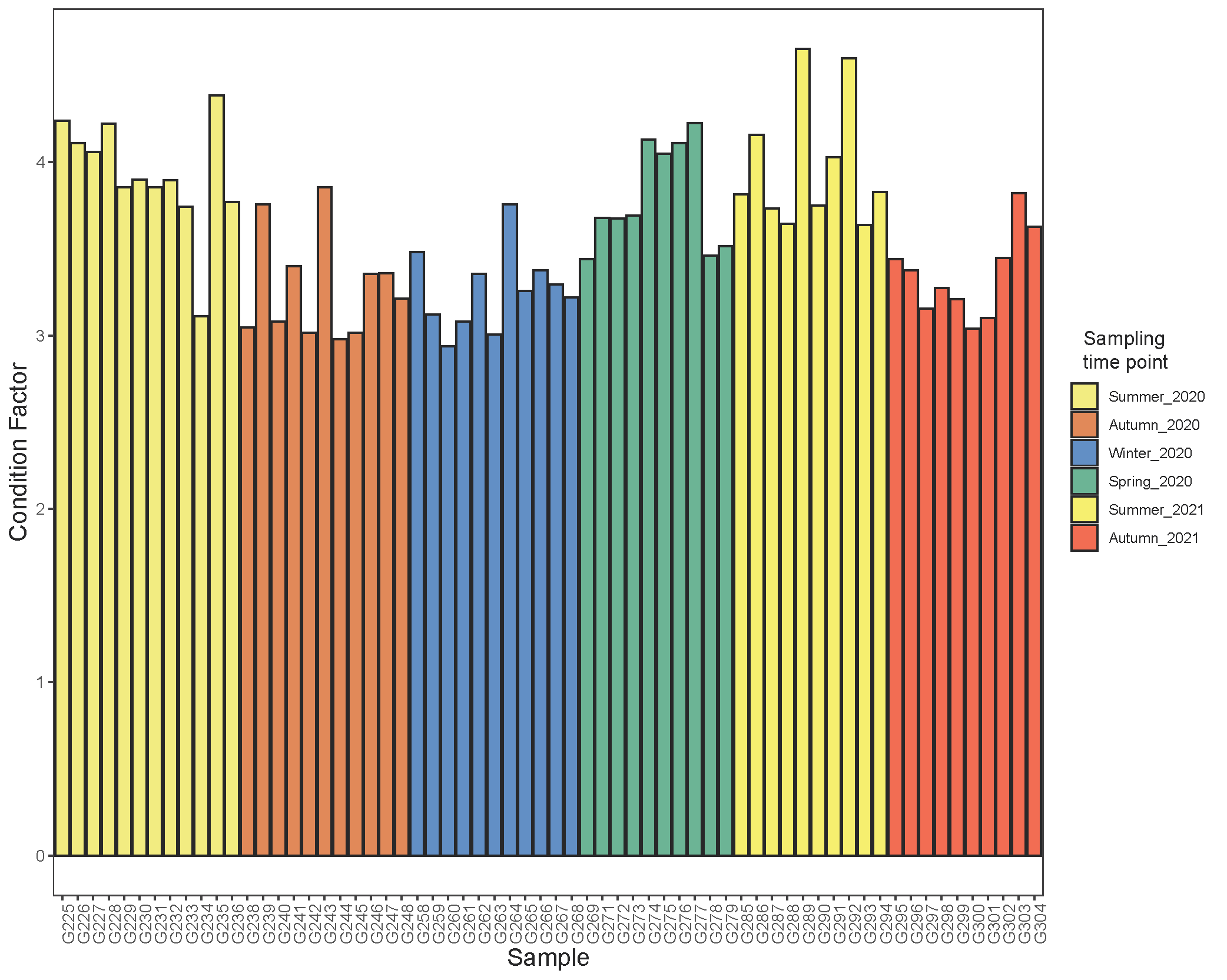


**Supplementary Figure 8.** Condition factor of individual fish coloured by sampling time points.


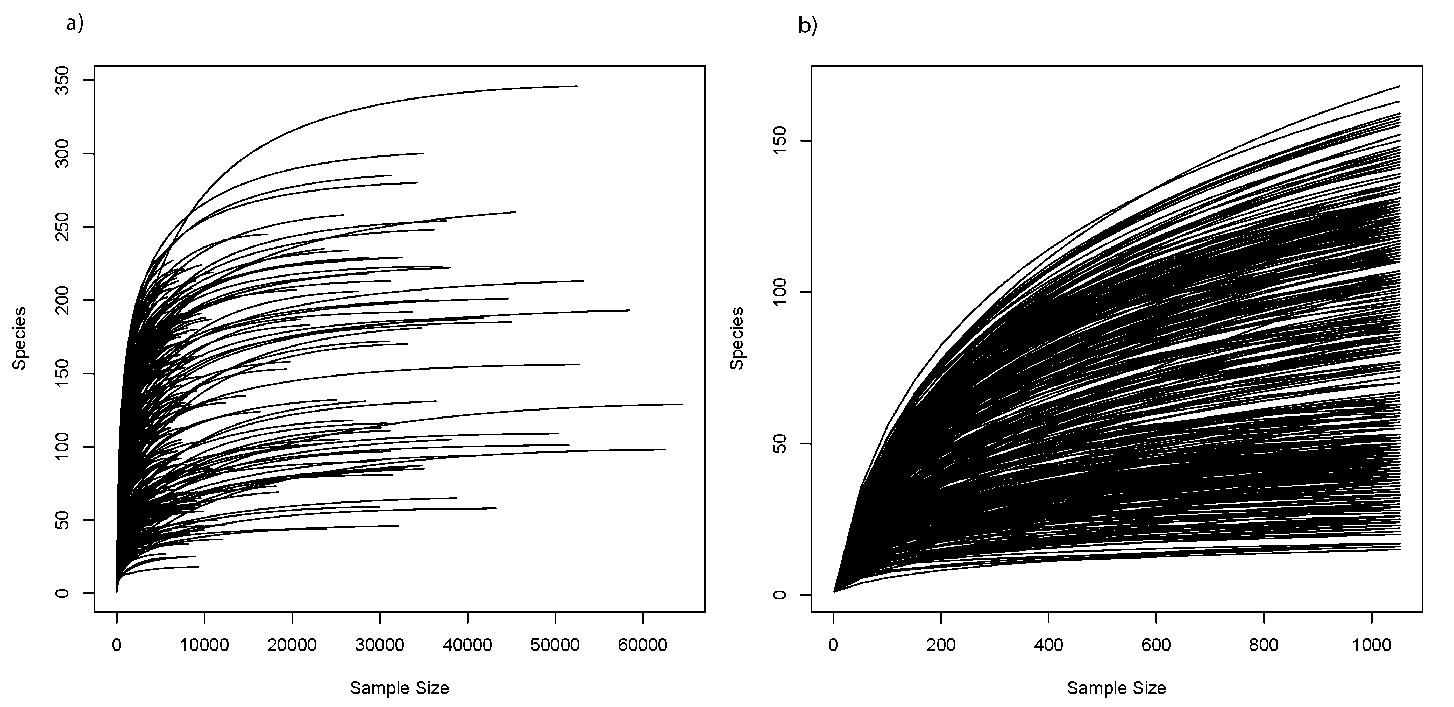


**Supplementary Figure 9.** Rarefaction sequencing curves. a) and b) illustrate species per sample size before and after rarefaction. Clear plateaus are observed indicating adequate richness was achieved at a depth of 1052.

**Supplementary Table 2.** Collection details for *K. sydneyanus.* GBI indicates Great Barrier Island and LBI indicates Little Barrier Island.

| **Fish no.** | **Species** | **Date & sampling time point** | **Location** | **Time** | **Standard length (cm)** | **Fork length (cm)** | **Total weight (g)** | **Gutted weight**  **(g)** | **Sex** | **SW Temp** °C |
| --- | --- | --- | --- | --- | --- | --- | --- | --- | --- | --- |
| G225 | *K. sydneyanus* | 14.01.20  Summer | Separation point, GBI | 1130 | 470 | 535 | 4400 | 3000 | Female | 18.5 |
| G226 | *K. sydneyanus* | 14.01.20  Summer | Separation point, GBI | 1250 | 440 | 500 | 3500 | 2600 | Male | 18.5 |
| G227 | *K. sydneyanus* | 14.01.20  Summer | Separation point, GBI | 1433 | 450 | 520 | 3700 | 2600 | Female | 18.5 |
| G228 | *K. sydneyanus* | 14.01.20  Summer | Separation point, GBI | 1452 | 510 | 580 | 5600 | 4050 | Female | 18.5 |
| G229 | *K. sydneyanus* | 14.01.20  Summer | Separation point, GBI | 1556 | 385 | 445 | 2200 | 1700 | Male | 18.5 |
| G230 | *K. sydneyanus* | 14.01.20  Summer | Nelson Island, GBI | 1730 | 430 | 500 | 3100 | 2450 | Male | 18.5 |
| G231 | *K. sydneyanus* | 14.01.20  Summer | Nelson Island, GBI | 1800 | 485 | 551 | 4400 | 3450 | Female | 18.5 |
| G232 | *K. sydneyanus* | 14.01.20  Summer | Nelson Island, GBI | 1830 | 450 | 510 | 3550 | 2750 | Female | 18.5 |
| G233 | *K. sydneyanus* | 14.01.20  Summer | Nelson Island, GBI | 1840 | 445 | 515 | 3300 | 2600 | Female | 18.5 |
| G234 | *K. sydneyanus* | 15.01.20 Summer | Nelson Island, GBI | 900 | 540 | 600 | 4900 | 3800 | Female | 18.5 |
| G235 | *K. sydneyanus* | 15.01.20 Summer | Nelson Island, GBI | 909 | 475 | 540 | 4700 | 3500 | Female | 18.5 |
| G236 | *K. sydneyanus* | 15.01.20 Summer | Nelson Island, GBI | 945 | 510 | 580 | 5000 | 4000 | Male | 18.5 |
| G238 | *K. sydneyanus* | 15.05.20 Autumn | Nelson Island, GBI | 1014 | 540 | 595 | 4800 | 3800 | Male | 17.5 |
| G239 | *K. sydneyanus* | 15.05.20 Autumn | Nelson Island, GBI | 1020 | 330 | 375 | 1350 | 1100 | Male | 17.5 |
| G240 | *K. sydneyanus* | 15.05.20 Autumn | Nelson Island, GBI | 1056 | 470 | 525 | 3200 | 2600 | Male | 17.5 |
| G241 | *K. sydneyanus* | 15.05.20 Autumn | Nelson Island, GBI | 1100 | 490 | 550 | 4000 | 3200 | Male | 17.5 |
| G242 | *K. sydneyanus* | 15.05.20 Autumn | Nelson Island, GBI | 1150 | 490 | 555 | 3550 | 2900 | Male | 17.5 |
| G243 | *K. sydneyanus* | 15.05.20 Autumn | Nelson Island, GBI | 1150 | 485 | 550 | 4400 | 3350 | Female | 17.5 |
| G244 | *K. sydneyanus* | 15.05.20 Autumn | Nelson Island, GBI | 1150 | 510 | 570 | 3950 | 3200 | Female | 17.5 |
| G245 | *K. sydneyanus* | 15.05.20 Autumn | Nelson Island, GBI | 1240 | 490 | 555 | 3550 | 2900 | Female | 17.5 |
| G246 | *K. sydneyanus* | 15.05.20 Autumn | Nelson Island, GBI | 1300 | 490 | 545 | 3950 | 3050 | Male | 17.5 |
| G247 | *K. sydneyanus* | 15.05.20 Autumn | Nelson Island, GBI | 1315 | 530 | 600 | 5000 | 3950 | Male | 17.5 |
| G248 | *K. sydneyanus* | 15.05.20 Autumn | Nelson Island, GBI | 1420 | 545 | 610 | 5200 | 3900 | Female | 17.5 |
| G258 | *K. sydneyanus* | 24.07.20 Winter | Nelson Island, GBI | 1017 | 480 | 545 | 3850 | 3250 | Male | 14.8 |
| G259 | *K. sydneyanus* | 24.07.20 Winter | Nelson Island, GBI | 1027 | 445 | 508 | 2750 | 2250 | Female | 14.8 |
| G260 | *K. sydneyanus* | 24.07.20 Winter | Nelson Island, GBI | 1040 | 485 | 540 | 3350 | 2800 | Female | 14.8 |
| G261 | *K. sydneyanus* | 24.07.20 Winter | Nelson Island, GBI | 1050 | 475 | 530 | 3300 | 2750 | Male | 14.8 |
| G262 | *K. sydneyanus* | 24.07.20 Winter | Nelson Island, GBI | 1110 | 490 | 550 | 3950 | 3300 | Female | 14.8 |
| G263 | *K. sydneyanus* | 24.07.20 Winter | Catherine Bay, GBI | 1230 | 550 | 600 | 5000 | 4100 | Female | 14.8 |
| G264 | *K. sydneyanus* | 24.07.20 Winter | Catherine Bay, GBI | 1240 | 440 | 505 | 3200 | 2600 | Female | 14.8 |
| G265 | *K. sydneyanus* | 24.07.20 Winter | Catherine Bay, GBI | 1248 | 530 | 595 | 4850 | 3850 | Female | 14.8 |
| G266 | *K. sydneyanus* | 24.07.20 Winter | Catherine Bay, GBI | 1320 | 485 | 545 | 3850 | 3200 | Female | 14.8 |
| G267 | *K. sydneyanus* | 24.07.20 Winter | Catherine Bay, GBI | 1428 | 515 | 575 | 4500 | 3500 | Female | 14.8 |
| G268 | *K. sydneyanus* | 24.07.20 Winter | Catherine Bay, GBI | 1422 | 475 | 530 | 3450 | 2900 | Male | 14.8 |
| G269 | *K. sydneyanus* | 14.10.20  Spring | Sugar loaf rocks, LBI | 1130 | 460 | 520 | 3350 | 2700 | Female | 15.7 |
| G271 | *K. sydneyanus* | 14.10.20 | Ahuriri rock, GBI | 1410 | 385 | 450 | 2100 | 1750 | Male | 15.7 |
| G272 | *K. sydneyanus* | Spring | Ahuriri rock, GBI | 1425 | 450 | 520 | 3350 | 2700 | Female | 15.7 |
| G273 | *K. sydneyanus* | 14.10.20 | Ahuriri rock, GBI | 1450 | 510 | 570 | 4900 | 3600 | Female | 15.7 |
| G274 | *K. sydneyanus* | Spring | Ahuriri rock, GBI | 1536 | 390 | 450 | 2450 | 2000 | Female | 15.7 |
| G275 | *K. sydneyanus* | 14.10.20 | Ahuriri rock, GBI | 1550 | 440 | 510 | 3450 | 2750 | Female | 15.7 |
| G276 | *K. sydneyanus* | Spring | Ahuriri rock, GBI | 1610 | 440 | 520 | 3500 | 2900 | Male | 15.7 |
| G277 | *K. sydneyanus* | 14.10.20 | Ahuriri rock, GBI | 1700 | 440 | 510 | 3600 | 2750 | Male | 15.7 |
| G278 | *K. sydneyanus* | Spring | Ahuriri rock, GBI | 1725 | 405 | 470 | 2300 | 1950 | Male | 15.7 |
| G279 | *K. sydneyanus* | 15.10.20 Spring | Catherine Bay, GBI | 920 | 470 | 525 | 3650 | 2950 | Male | 15.7 |
| G285 | *K. sydneyanus* | 27.01.21 Summer | Catherine Bay, GBI | 1155 | 320 | 365 | 1250 | 1000 | Male | 20.4 |
| G286 | *K. sydneyanus* | 27.01.21 Summer | Catherine Bay, GBI | 1229 | 315 | 365 | 1300 | 1050 | female | 20.4 |
| G287 | *K. sydneyanus* | 27.01.21 Summer | Ahuriri bay, GBI | 1414 | 510 | 575 | 4950 | 3650 | Female | 20.4 |
| G288 | *K. sydneyanus* | 27.01.21 Summer | Ahuriri bay, GBI | 1426 | 420 | 470 | 2700 | 2100 | Female | 20.4 |
| G289 | *K. sydneyanus* | 27.01.21 Summer | Ahuriri bay, GBI | 1506 | 430 | 480 | 3700 | 2800 | Female | 20.4 |
| G290 | *K. sydneyanus* | 27.01.21 Summer | Ahuriri bay, GBI | 1558 | 360 | 410 | 1750 | 1400 | Male | 20.4 |
| G291 | *K. sydneyanus* | 27.01.21 Summer | Ahuriri bay, GBI | 1623 | 310 | 350 | 1200 | 900 | adult fish but sexually immature | 20.4 |
| G292 | *K. sydneyanus* | 28.01.21 Summer | Miner's head, GBI | 939 | 510 | 575 | 6100 | 4400 | Female | 20.4 |
| G293 | *K. sydneyanus* | 28.01.21 Summer | Miner's head, GBI | 943 | 485 | 545 | 4150 | 3000 | Male | 20.4 |
| G294 | *K. sydneyanus* | 28.01.21 Summer | Ahuriri bay, GBI | 1100 | 465 | 525 | 3850 | 2950 | Female | 20.4 |
| G295 | *K. sydneyanus* | 19.04.21 Autumn | Catherine Bay, GBI | 1049 | 490 | 545 | 4050 | 3000 | Female | 20.2 |
| G296 | *K. sydneyanus* | 19.04.21 Autumn | Catherine Bay, GBI | 1125 | 485 | 540 | 3850 | 2950 | Female | 20.2 |
| G297 | *K. sydneyanus* | 19.04.21 Autumn | Catherine Bay, GBI | 1201 | 485 | 535 | 3600 | 2750 | Female | 20.2 |
| G298 | *K. sydneyanus* | 19.04.21 Autumn | Catherine Bay, GBI | 1250 | 470 | 525 | 3400 | 2650 | Female | 20.2 |
| G299 | *K. sydneyanus* | 19.04.21 Autumn | Catherine Bay, GBI | 1405 | 480 | 540 | 3550 | 2750 | Female | 20.2 |
| G300 | *K. sydneyanus* | 19.04.21 Autumn | Catherine Bay, GBI | 1430 | 525 | 590 | 4400 | 3450 | Female | 20.2 |
| G301 | *K. sydneyanus* | 19.04.21 Autumn | Catherine Bay, GBI | 1500 | 555 | 620 | 5300 | 3800 | Female | 20.2 |
| G302 | *K. sydneyanus* | 19.04.21 Autumn | Catherine Bay, GBI | 1544 | 520 | 590 | 4850 | 3700 | Female | 20.2 |
| G303 | *K. sydneyanus* | 19.04.21 Autumn | Catherine Bay, GBI | 1544 | 455 | 515 | 3600 | 2400 | Male | 20.2 |
| G304 | *K. sydneyanus* | 19.04.21 Autumn | Catherine Bay, GBI | 1620 | 520 | 585 | 5100 | 3900 | Female | 20.2 |
